# Splitting aptamers and nucleic acid enzymes for the development of advanced biosensors

**DOI:** 10.1093/nar/gkaa132

**Published:** 2020-02-29

**Authors:** Mégane Debiais, Amandine Lelievre, Michael Smietana, Sabine Müller

**Affiliations:** 1 Institut des Biomolécules Max Mousseron, University of Montpellier, CNRS, ENCSM, Montpellier, France; 2 University Greifswald, Institute for Biochemistry, Greifswald, Germany

## Abstract

In analogy to split-protein systems, which rely on the appropriate fragmentation of protein domains, split aptamers made of two or more short nucleic acid strands have emerged as novel tools in biosensor set-ups. The concept relies on dissecting an aptamer into a series of two or more independent fragments, able to assemble in the presence of a specific target. The stability of the assembled structure can further be enhanced by functionalities that upon folding would lead to covalent end-joining of the fragments. To date, only a few aptamers have been split successfully, and application of split aptamers in biosensing approaches remains as promising as it is challenging. Further improving the stability of split aptamer target complexes and with that the sensitivity as well as efficient working modes are important tasks. Here we review functional nucleic acid assemblies that are derived from aptamers and ribozymes/DNAzymes. We focus on the thrombin, the adenosine/ATP and the cocaine split aptamers as the three most studied DNA split systems and on split DNAzyme assemblies. Furthermore, we extend the subject into split light up RNA aptamers used as mimics of the green fluorescent protein (GFP), and split ribozymes.

## INTRODUCTION

**Figure 1.**
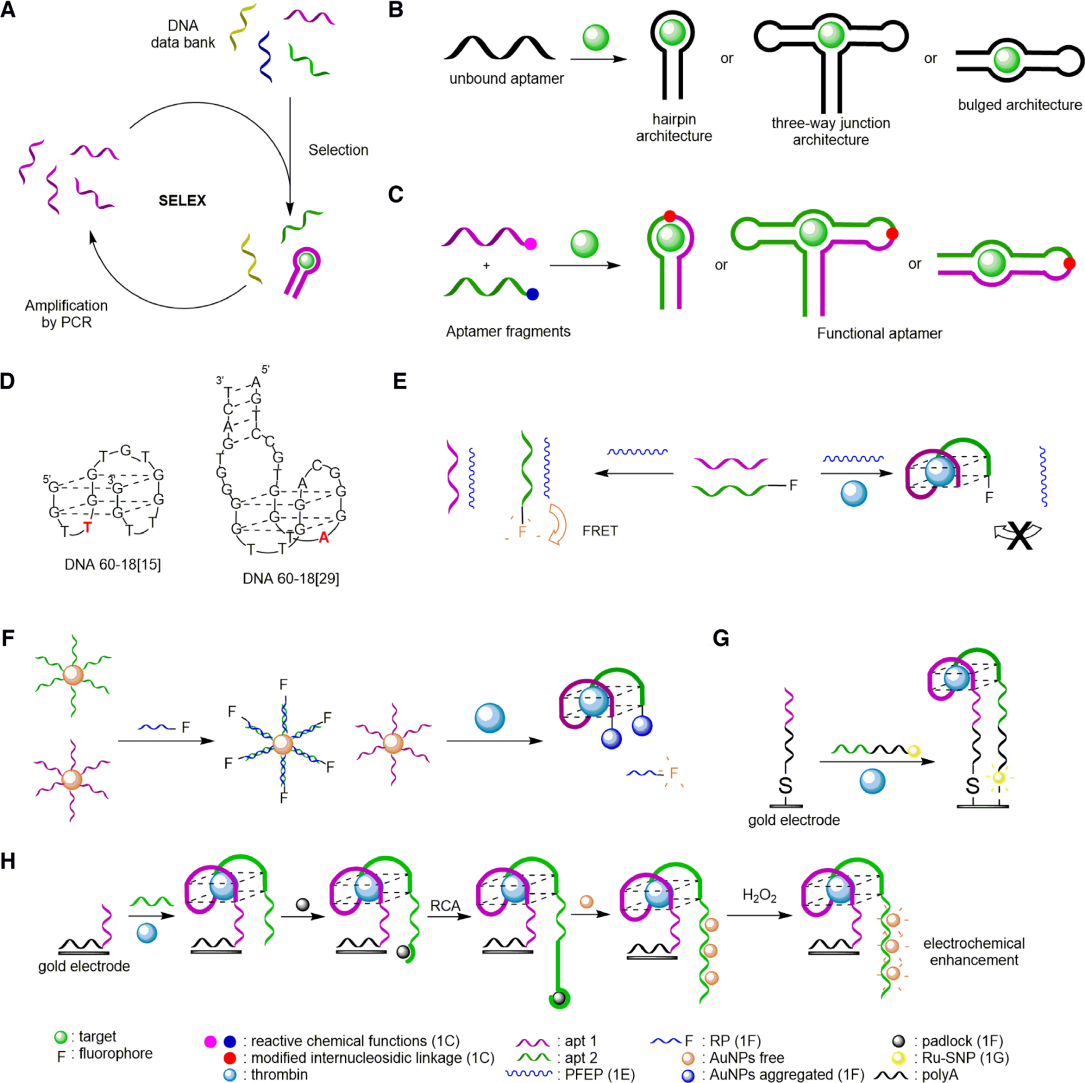
General concept and thrombin detection by aptamers and split aptamers. (**A**) *In vitro* selection of aptamers by SELEX (13–15). (**B**) Conformational change of aptamers in presence of the target (18). (**C**) General scheme of the split aptamer concept (18). (**D**) Three-dimensional structure of the two main DNA aptamers with the nucleotide that directs the aptamer to one of the binding sites marked in red (39, 44). (**E**) Thrombin detection by conjugation of two split aptamers with PFEP (50). (**F**) Thrombin detection by fluorimetry and colorimetry (51). (**G**) Thrombin detection by ECL using an Ru-SNP complex (58). (**H**) Thrombin detection by ECL based on RCA (59).

Besides the well-known roles of DNA and RNA as sources and carriers of genetic information (1,2), nucleic acid sequences are known to adopt a large variety of topologies that can be employed in bioanalytical sciences (3). After the discovery of ribozymes by the Cech and Altman laboratories (4,5), Breaker and Joyce described for the first time in 1994 that also single‐stranded DNA sequences can exhibit enzymatic activity (6), an attribute that is currently extensively used for sensing and nanobiotechnological applications (7–10). Similarly, since their discovery in the early 1990s, aptamers being single‐stranded RNA or DNA structures with the ability of binding molecules with high affinity and specificity, have stimulated tremendous interest in biology and medicine (11,12). Aptamers can be obtained by *in vitro* selection (Figure 1A), following the classical methodology of Systematic Evolution of Ligands by Exponential Enrichment (SELEX), first introduced by Tuerk and Gold in 1990 (13–15). For selection of deoxyribozymes (DNAzymes) and ribozymes, the classical SELEX process needs to be extended from selection of just binders to more complex functional screening, although iterative cycles of selection and amplification are retained. DNA- or RNA-based aptamers are able to interact specifically with a large variety of targets such as small molecules, ions, enzymes or proteins employing all kinds of non-covalent interactions. The first aptamers were revealed in 1990 by Ellington and Szostak (16) and composed exclusively of RNA sequences. In 1992, Bock *et al.* introduced also DNA single-stranded aptamers (17).

The most common architecture of aptamers is the hairpin or hairpin-like structure, in which the target binding site is situated in the loop region. There are also three-way junction topologies composed of three DNA stems with the target binding site located at the branch point, as well as bulged structures with the target binding site at an internal loop (Figure 1B). Upon binding of the target molecule, most aptamers undergo a conformational change, thus following the induced fit model. In all cases, the well-defined three-dimensional structure is responsible for the observed high specificity (18,19). Easy to synthesize, aptamers display very attractive features for target recognition (20–22) with therapeutic (11,20,23), diagnostic (24) and analytical applications (25). Moreover, once selected, aptamers are easy to modify in order to increase their stability against nucleases (26), or to conjugate with a large variety of partners (fluorescent tags, specific probes, lipophilic or cationic biomolecules, etc.) (20,27,28). Their ability to switch from a random coil to an organized conformation in the presence of a target has thus been exploited efficiently in fluorescent sensors (21,29,30). Although being very attractive, aptamers are not devoid of limitations (31). Degradation by nucleases can be limited by the incorporation of modified nucleotides, but the number of negative charges (generally from 20 to 60) hampers their intracellular delivery. In addition, long aptamers can form unfavorable secondary structures that could interact with complex matrixes and may lead to false positive or nonspecific signals.

In analogy to split protein systems, which rely on the appropriate fragmentation of protein domains (32), split aptamers made of two or more short nucleic acid strands emerged recently to engineer systems easier to synthesize and with less negative charges per strand (33). In this concept, a defined aptamer is cut into a series of two or more independent and non-functional fragments, which are able to assemble selectively in the presence of the target. The strong interactions between the ligand and the aptamer-based structure modify the thermodynamic equilibrium and promote the assembly of the fragments (Figure 1C). The stability of the assembled structure can be further enhanced by decorating individual strands with functionalities that upon folding would lead to covalent end-joining of the fragments. Assembly brings the two reactive functionalities in close proximity and thus allows, upon chemical activation, the formation of an additional natural or modified internucleosidic linkage. To date, within the >100 small-molecule-binding aptamers that have been discovered, only a few have been split successfully (34,35). The difficulty to design split aptamers arises from the necessary 3D structure of the parent aptamer, which can be highly perturbed if the division is made at the wrong site, like for example in a hairpin binding pocket. If the target molecule binds in the stem region, aptamers are easier to divide. As for three-way junction-based aptamers, the arms can be easily divided into short fragments, and yet preserving the interaction with the target (18).

In this review, we first focus on the three most studied DNA split aptamers obtained so far from a parent aptamer, namely the thrombin, the adenosine/ATP and the cocaine split aptamers. Then, we give some examples of split DNAzyme–aptamer assemblies. Lastly, we extend the subject to RNA aptamers used as mimics of the green fluorescent protein (GFP), which are the split Spinach and the split Broccoli systems, and further on to split ribozymes.

## HUMAN α-THROMBIN SPLIT APTAMER

### Properties

Thrombin, also called coagulation factor, is a trypsin-like serine protease that plays an essential role in thrombosis and haemostasis (36,37). It exists in several forms, but only the α-thrombin seems to be physiologically important, whereas the β- and γ-thrombin are much less effective (37,38). Human α-thrombin is thus a major target for therapy of anticoagulation and cardiovascular diseases.

The first inhibitor to human thrombin, isolated by Bock *et al.* in 1992 by *in vitro* selection (17), was also the first DNA aptamer to be discovered. After five rounds of selection, 32 oligonucleotide sequences were obtained, capable of binding to thrombin. All of them were composed of 60 nucleotides of random sequence with a conserved region of 14–17 nucleotides (5′-GGNTGGN_2–5_GGNTGG-3′, N being a variable nucleotide), responsible of binding and inhibiting thrombin. The sequence 5′-GGTTGGTGTGGTTGG-3′, called **60-18[15]** (Figure 1D), was determined to have the highest thrombin affinity and inhibitory activity in the presence of potassium ions (39–41). NMR spectroscopy and X-ray crystallography studies revealed that the thrombin aptamer forms a highly compact structure, composed of an intramolecular G-quadruplex with an antiparallel orientation and a chair-like conformation (39,42).

In 1995, Macaya *et al.* reported a DNA oligonucleotide obtained by the same process with a quadruplex/duplex structure composed of the previous sequence and four to seven added base-pairs flanking the G-quadruplex motif, which allows to considerably increase the affinity (*K*_d_ values of 10 to 25 nM versus 100 nM for **DNA60-18[15]**) (43). Then, in 1997, Tasset *et al.* reported a 29-mer DNA oligonucleotide, called **60-18[29]**, obtained by nitrocellulose filter partition, having a minimal sequence of 5′-NNNCCGTGGTAGGGNAGG^A^/_T_TGGGGTGN’N’N’-3′, where N and N’ are complementary variable nucleotides, and a *K*_d_ value of 0.5 nM (44). This aptamer has also a quadruplex/duplex structure (Figure 1D) but is potassium-independent and, in contrast to previously reported aptamers that interact with the fibrinogen-recognition exosite (FRE), binds to the electropositive heparin-binding exosite. This change of binding site was attributed to the orientation of the G-quadruplex and its fourth nucleotide (red residue in Figure 1D), determining the selectivity of the binding site to the aptamer (44–46).

### Fluorescent biosensor

Highly sensitive aptamer-based fluorescent sensors for thrombin have been widely described (47–49). In order to develop more specific biosensors, aptamers were replaced by split aptamer fragments, that are shorter, less secondary structured, and conjugated with different molecules to amplify the signal. Thus, in 2014, Liu *et al.* designed a new fluorescence-based strategy by using a water-soluble polycationic polymer, (poly{[9,9-bis(6′-(*N*,*N*,*N*-diethylmethylammonium)hexyl)-2,7-fluorenylene ethynylene]-alt-co-[2,5-bis(3′-(*N*,*N*,*N*-diethylmethylammonium)-1′-oxapropyl)-1,4-phenylene]} tetraiodide, PFEP), having a high fluorescence emission, and two fragments of the 15-mer thrombin binding DNA aptamer, one of them being labelled with fluorescein (Figure 1E) (50). In the absence of thrombin, the two fragments bind to the polymer *via* strong electrostatic interactions and lead to an intense Förster resonance energy transfer (FRET) phenomenon. However, the presence of thrombin triggers the formation of the G-quadruplex structure that decreases the interaction between the polymer and the fluorophore and results in a significant reduction of the FRET signal. Compared with the full-length aptamer, this split aptamer-based sensing strategy is isothermal and increases the possibility of forming the G-quadruplex upon thrombin addition, and thus the sensitivity (LOD = 2 nM). A similar approach using identical split aptamer fragments adsorbed on graphene oxide (GO) (51) took advantage of the ability of GO to adsorb single-stranded DNA and of GO fluorescence-quenching properties associated with high electronic and thermal conductivities (52,53). In the absence of thrombin the two fragments are adsorbed on GO *via* non-covalent π–π stacking interactions, resulting in quenching of the fluorescence. Addition of thrombin triggers the formation of the G-quadruplex structure and restores the fluorescence with a sensitivity in the nanomolar range (LOD = 1 nM) that could not be achieved with the intact aptamer.

More recently, a highly selective and sensitive method that combines fluorimetry and colorimetry has been developed (54). The strategy uses two fragments of the thrombin aptamer anchored on gold nanoparticles (AuNPs) by poly-adenosine (polyA) DNA extensions (Figure 1F). One of the fragments is caged in a duplex with a complementary strand containing a fluorescent reporter probe (RP). In the absence of thrombin, the hybridization of RP with its complementary split aptamer fragment leads to quenching of the fluorophore by the AuNPs (55). At the same time, owing to the high electrostatic repulsion, AuNPs cannot aggregate, mirrored in a wine red color of the solution. The presence of thrombin triggers the formation of the G-quadruplex structure and the aggregation of AuNPs, while simultaneously releasing the RP. This is monitored by a change of color of the solution from wine red to blue (LOD = 0.45 nM) and by a fluorescence increase of the RP (LOD = 0.16 nM). Interestingly, this dual mode detection strategy can discriminate thrombin from other analogues in human serum samples.

### Electrochemiluminescent biosensor

Electrochemiluminescence (ECL) is a process that can emit light when species generated at the surface of electrodes undergo high-energy electron-transfer. ECL has received increasing attention in biomolecule analysis due to its efficiency, high sensitivity and wide linear range. Ru(bpy)_3_^2+^ is the most used ECL reagent and has been employed in conjunction with silica nanoparticles to develop an aptamer-based ECL sensor for thrombin detection (Ru-SNPs) (56–58). An illustration is given by the work of Lin *et al.*, who split the 15-nucleotide thrombin aptamer into two different fragments: the first fragment was modified with an alkanethiol at its 5′-extremity and immobilized on a gold electrode, and the second one was coupled with Ru-SNPs at the 3′-extremity *via* an amide link (58). In the absence of thrombin the two fragments are dissociated resulting in a weak ECL signal whereas addition of thrombin triggers the formation of the G-quadruplex structure and provokes the attachment of Ru-SNPs onto the electrode surface, an event that can be monitored by ECL measurements (Figure 1G). Apart from being highly sensitive (LOD = 0.2 pM), the method explores new sensing strategies of split aptamer fragments.

### Electrochemical detection

Electrochemical aptasensors based on split systems have also been described. Recently, the Fan laboratory developed a novel electrochemical label-free strategy for thrombin detection (59). The concept was based on the well-known affinity of polyA for gold nanoparticles (55,60,61). The authors exploited these polyA-Au interactions by designing a split system from the above-mentioned 15-mer DNA sequence (17). Using a polyA sequence as an anchoring block allowed the immobilization of the first fragment on a gold electrode surface *via* polyA-Au interactions (Figure 1H). The second fragment was also extended by a short sequence containing several adenosine residues. The addition of thrombin and the second split aptamer fragment promoted the formation of the G-quadruplex structure, and the extended part of the second fragment was then, upon addition of a padlock probe, subjected to rolling circle amplification (RCA), leading to long DNA sequences containing repeating A_6_ sequences. AuNPs were then added and adsorbed on the surface of the aptasensor by polyA-AuNPs interactions. Finally, upon addition of H_2_O_2_, electrocatalytic reduction of the AuNPs was monitored. This aptasensor showed high selectivity and remarkable sensitivity with a LOD of 35 fM in a linear detection range from 0.1 pM to 10 nM, and it was demonstrated to be as efficient as commercial ELISA assays on real sample analysis.

## ADENOSINE AND ADENOSINE TRIPHOSPHATE SPLIT APTAMER

### Properties

**Figure 2.**
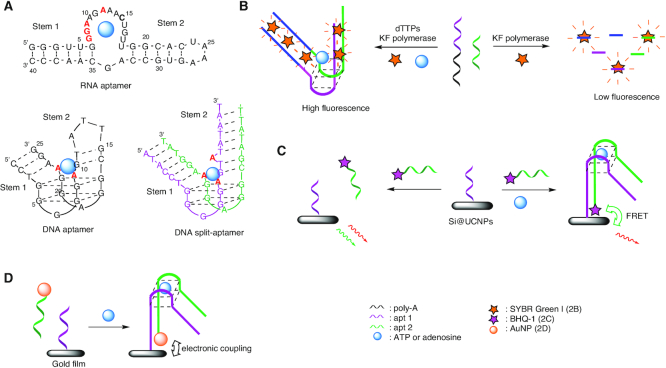
ATP and adenosine detection by aptamers and split aptamers. (**A**) RNA and DNA aptamers and DNA split aptamer (33,64). (**B**) Label-free method with KF polymerase and SYBR Green I for split aptamer based adenosine detection (65). (**C**) ATP detection by sandwich-FRET assay (66). (**D**) Adenosine detection by SPR with AuNPs (82).

Adenosine triphosphate (ATP), discovered in parallel by Lohmann, Fiske and Subbarow in 1929 (62,63), is implied in many biological and cell signalling processes. ATP dysregulations are associated with various disorders, which renders its detection particularly important. The first ATP-aptamer was isolated by Sassanfar and Szostak in 1993 by *in vitro* selection and consisted of a 40-mer RNA having a 11-nucleotides loop sequence and a *K*_d_ of 0.7–8 μM, depending on the specific salt and Mg^2+^ concentrations (Figure 2A) (64). A couple of years later, Szostak and Huizenga isolated and characterized a 25-nucleotides long DNA aptamer for adenosine and ATP composed of two stacked G-quartets, with a *K*_d_ of 6 μM (Figure 2A) (33). Although not defining it as a split system, the authors showed that the aptamer could be divided into two fragments by removing the loop that closes stem 2 and by extending each fragment with additional nucleotides to compensate the loss of stability. Neither of these fragments could bind ATP or adenosine alone, but when hybridized, target binding was reactivated (Figure 2A). To the best of our knowledge, this article reports the very first experiment in the field of split aptamers, although no LOD was reported at that time.

### Fluorescent biosensor

The adenosine DNA aptamer was cut into two halves to develop a label-free method for selective detection of adenosine. A 21 nucleotides homo dA sequence was added to the first fragment to serve as a template for Klenow fragment polymerase (KF polymerase, Figure 2B) (65). Moreover, both fragments were slightly modified at their extremities to have complementary sequences, which however, are too short to be annealed at ambient temperature. SYBR Green I was selected for its ability to exhibit high fluorescence enhancement upon binding to dsDNA. When mixed together, both fragments do not form a sufficiently stable initiation complex for Klenow polymerization, and thus are rapidly digested by the exonuclease activity of KF polymerase. Hence, no dsDNA is available for SYBR Green binding and the fluorescence intensity response is very low. When adenosine is present, reassembly of the two ssDNA fragments is supported by adenosine binding, thus generating a primer-template complex for the DNA polymerase reaction that leads to the formation of a duplex DNA structure and a strong increase of the dye's emission intensity with a LOD of 12 μM. Although the detection limit is the same as found for the full-length aptamer-based adenosine sensors, the method presents the advantage of avoiding fragment labeling.

He *et al.* used a sandwich-type FRET assay to increase the selectivity by conjugating one of the aptamer fragments to the dye BHQ-1, and the other to upconverting nanoparticles containing a silica coating (Si@UCNPs) that is used as an energy donor (Figure 2C) (66). In the absence of the target molecule, red and green fluorescence of the Si@UCNPs is observed, whereas addition of ATP triggers the formation of a sandwich complex allowing FRET between the Si@UCNPs and the BHQ-1, and therefore quenching of the green fluorescence. Moreover, this system allows to discriminate ATP from its CTP, UTP and GTP analogues with a LOD of 1.7 μM.

More recently, an ATP split aptamer platform was designed for the detection of adenosine deaminase activity *via* FRET from AuNPs and gold nanoclusters-linked aptamer fragments (67). The enzymatic activity was monitored by the transformation of ATP into inosine triphosphate (which has no affinity to the aptamer) with a LOD of 0.72 U^.^L^−1^, which compares favorably to other reported techniques (68).

The ATP aptamer was also split into two fragments labelled with pyrenes and evaluated in the presence of γ-cyclodextrin (γ-CD) that served as a space modulator (69). The system allows to detect ATP within a linear range from 5.0 to 50 μM and a LOD of 80 nM in buffer solution, and 0.5 μM in blood serum. More recently, Zhang *et al.* developed an ultrasensitive ATP detection method based on dual-color fluorescence co-localization of split aptamers (70). According to the previously reported design (69) the two fragments were labelled with Cy3 and Cy5, respectively. The Cy3 labelled sequence was immobilized onto a surface through specific streptavidin/biotin interactions, whereas the Cy5 labelled fragment was free in solution. Upon addition of ATP the aptamer target complex is formed and the two strands become co-localized, which is mirrored in dual-color fluorescence. This assay can detect ATP in a linear range from 1 pM to 5 nM with a LOD of 100 fM. By distinguishing the kinetic signature of the fragments, the strategy avoids false positive detection and significantly increases the sensitivity as compared to previously reported methods. Given the interest of this type of systems, many recent studies report new designs of split aptamers able to detect ATP or adenosine by fluorescence (71–74).

### Sandwich assays and surface plasmon resonance (SPR)

A method widely used for studying biomolecular interactions is SPR spectroscopy that allows to detect refractive index (RI) changes on the surface of metal nanoparticles when the ligand binds to the receptor (75–77). This phenomenon is characterized by a specific absorption band, which is shifted as a function of the dielectric environment on the nanoparticle surface. However, while this label-free and real-time technique is very useful, it is challenging to apply the method to small molecules, because the signal is proportional to a change of molecular weight. Still, the method has been applied for sensing adenosine and/or ATP. A thermodynamic aptasensor was developed, in which the two fragments are grafted on the surfaces of the SPR sensor and AuNPs, respectively. The hybridization of the two sequences and the determination of their melting temperature was monitored by a home-made thermo-regulated SPR chamber. In the presence of adenosine, the aptamer/target complex is formed, leading to an increase of the melting-temperature, which was found to be dependent on adenosine concentration (78). Thanks to the signal amplification provided by the AuNP functionalization, this approach enabled to reach a LOD 200 times lower than with the native aptamer (LOD = 30 nM versus 6 μM). Compared to fluorescence-based biosensors, SPR biosensors do not require fluorescent labels, which can limit the background signal and are prone to bleaching. These advantages are illustrated by many recent reports dealing with SPR-based split aptamer biosensors (79–81).

Combination of SPR and nanoparticles allowed the development of a high-sensitive aptasensor for adenosine based on split aptamer fragments. This approach is illustrated by the study of Wang *et al.*, in which one fragment is immobilized at its 5′-terminus on a gold film, whereas the second fragment is conjugated to AuNPs (Figure 2D) (82). In the absence of adenosine, the two fragments are not able to form a stable duplex, and no resonance wavelength shift could be monitored. However, in presence of the target, the two single-strands assemble to form the adenosine–aptamer complex on the Au film, thus leading to a shift of the resonance wavelength. The LOD of this SPR biosensor was determined as 1.5 pM.

## COCAINE SPLIT APTAMER

### Properties

**Figure 3.**
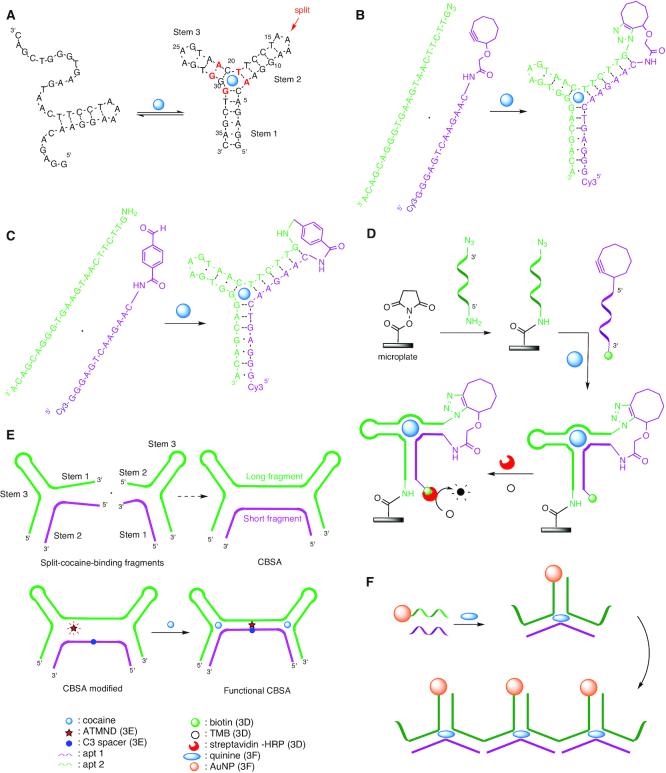
Cocaine detection by its aptamer and split aptamers. (**A**) Proposed secondary structure of the cocaine aptamer with nucleotides essential for cocaine binding marked in red, and the splitting position indicated by a red arrow (83). (**B**) DNA-templated click reaction controlled by cocaine binding (100). (**C**) DNA-templated reductive amination controlled by cocaine binding (102). (**D**) DNA-based analogue of ELISA for cocaine detection (103). (**E**) Design and modification of the CBSA by a C3 spacer to detect cocaine by formation of a functional CBSA (104). (**F**) Aptachain formation triggered by quinine (109).

The cocaine aptamer, discovered by Stojanovic *et al.* in the late 1990s (83), has been widely studied (35,84). This aptamer with a three-way junction architecture is composed of three helical parts. Stems 1 and 3 consist of six and four base pairs, respectively, including two noncanonical pairs, and stem 2 consists of five Watson–Crick base pairs (Figure 3A). Stem 2 is always double-stranded, while stems 1 and 3, which are in equilibrium between the single-stranded and double-stranded state, form a stable duplex only in the presence of cocaine at concentrations in the range of 10 μM to 1 mM (83,85). Studies were carried out to determine the optimal conditions for maximizing the affinity between the aptamer and the target. It appears that a pH value of 7.4 is necessary to simultaneously have the tertiary amine of the cocaine protonated and the phosphate backbone of the DNA deprotonated, which allows to considerably increase electrostatic interactions between the two entities. Three base pairs, namely G31/C6, G29/A21 and T19/A7, are indispensable for cocaine binding, because they directly interact with the target and are important for formation of the required secondary structure.

A simple method to monitor aptamer folding in the presence of cocaine is to functionalize the aptamer with a fluorophore and a quencher at its 5′- and 3′- extremities, respectively. In the presence of the target, the 5′- and 3′-end of the aptamer are brought in close proximity, leading to quenching of the fluorescence (LOD = 1 μM) (83,86–88). Other fluorescence-based systems have been described recently (89,90), and the different conformations have been studied by NMR or ITC (86,91,92). Another method to analyze cocaine binding is to divide the aptamer into two strands and to functionalize the fragments with fluorescent tags (83). Amplification strategies (93), electrochemical sandwich assays (94) and nanoparticles (95,96) were also developed for the detection of cocaine at low concentration. Since cocaine binds at the junction of the three helices, the construction of a split aptamer architecture is feasible by dividing the aptamer in the loop region of stem 2, where interactions between the two resulting strands and the small molecule will be less influenced.

### Click-chemistry

DNA can be used as a template to guide and promote chemical reactions owing to the Watson–Crick interaction of two strands that allows to bring the two reagents closer, increasing the effective molarity of the reactants and facilitate the reaction. Because of its biocompatible reaction conditions (efficient in aqueous media, rapid, high yielding and inoffensive by-products) (97) azide–alkyne cycloaddition (click chemistry) has been frequently applied to nucleic acid structures for applications in biology, nanotechnology or material sciences (98).

In most cases, the DNA-templated reaction is directed by the association between the two strands according to Watson-Crick affinity (99) but, in 2011, Heemstra and Sharma showed that the DNA-templated reaction can be controlled by small-molecule binding (100). To achieve this control, they split the cocaine aptamer at the loop region of stem 2 and modified one strand at its 3′-extremity with an azide, and the other at its 5′-extremity with a cycloalkyne. In the presence of cocaine, the two strands are brought together and the copper-free azide–alkyne cycloaddition can proceed, allowing to block the system in this conformation, in contrast to previous systems that are reversible (LOD = 1 μM–1 mM) (Figure 3B). Successful click reaction has been visualized by denaturing polyacrylamide gel electrophoresis (PAGE) using the Cy3 fluorophore at the 5′-end of the clicked aptamer for detection. Selectivity of the split aptamer ligation was studied by replacing cocaine with similar metabolites. The results showed a decreased binding capacity of all tested metabolites, except norcocaine, which has the most similar structure to cocaine. This result confirms earlier studies with the complete aptamer (101), showing that the methyl ester and the benzoyl group of cocaine are essential for recognition and binding, whereas the methyl group of the bridging nitrogen seems to be less important. In general, background click ligation independent of the ligand was found to take place to a significant extent, and therefore reductive amination was explored as an alternative chemistry. Using a 5′-amino and a 3′-aldehyde substrate allowed to detect cocaine at concentrations as low as 1 μM and quinine and quinidine down to 100 nM (Figure 3C) (102).

The DNA-templated reaction assisted by cocaine was used in a DNA-based analogue of the well-known sandwich enzyme-linked immunosorbent assay (ELISA), applicable to small-molecule detection (103). First, the capture strand modified by an azide at the 5′-end and an amine at the 3′-end reacts with a *N*-hydroxysuccinimide covered microplate to form an amide link (Figure 3D). Then, the detection strand modified at the 3′-end by a cyclooctyne and at the 5′-end by a biotin is added in the presence of cocaine to generate the split aptamer structure that will trigger ligation. Finally, a conjugate constituted of streptavidin and horse radish peroxidase (HRP) is added to bind to biotin and subsequently allows reaction with tetramethylbenzidine (TMB), resulting in a blue colored product. In this set-up, the detection of cocaine is sensitive (LOD = 1 μM) and easy, but the response was fivefold slower than observed in previous studies, where a strong dose-dependent ligation was obtained after 4 h (100). This observation may be explained by the attachment of the azide containing strand to the microplate, which presumably makes the azide less accessible. By replacing the aryl-less cyclooctyne (ALO) with dibenzoazacyclooctyne (DIBAC), known to be 240-fold more reactive, the reaction becomes faster. As mentioned above, in the original version, ligation was observed also in the absence of the small-molecule target, thus it appeared necessary to replace a GC base pair in stem 2 with a CC mismatch, and a GT wobble pair in stem 3 with a GC base pair, to decrease the affinity between the two strands. Moreover, by adding a linker between the microplate and the capture strand, the signal could be considerably increased, probably by easier access of the detection strand to the azide. This modified system proved able to detect cocaine at very low concentrations, 100 nM in buffer and 1 μM in human blood serum. All these parameters make it the so far most sensitive and fastest split aptamer analogue of sandwich ELISA.

### Cooperative-binding split aptamer

The split aptamer concept is advantageous in terms of easier synthesis and delivery of the nucleic acid strands constituting the aptamer, but on the other side harbors the risk of a decrease of target affinity and low sensitivity. This problem was addressed with a recently developed cooperative-binding split aptamer (CBSA) having two binding sites (104). The first cocaine-binding event stabilizes the structure of the split aptamer and facilitates binding of the second cocaine molecule at the second binding site, thus in the aggregate allowing to achieve higher sensitivity and target affinity. The system uses two different pairs of cocaine split aptamer fragments truncated in stems 1 and 2 (105). The CBSA was constructed by combining stem 1 of the first split aptamer with stem 2 of the second to form one long and one short fragment that can assemble in the presence of the target (Figure 3E). Then, the short fragment sequence was modified by replacing an adenosine at position 10 with a C3 spacer in order to form an abasic site that serves to monitor the assembly by complexation with 2-amino-5,6,7-trimethyl-1,8-naphtyridine (ATMND). Indeed, ATMND is fluorescent in solution, but when the two strands are assembled, the fluorescence signal is quenched by the C3 spacer. The system was found to detect cocaine with a LOD of 50 nM in buffer or in 10% diluted saliva. In 2018, a similar system based on CBSA has been developed using enzyme-assisted target recycling to amplify signals, allowing to detect cocaine at 1 μM in 50% urine (106).

### Aptachain

The aptachain concept relies on splitting an aptamer into two overlapping strands able to assemble upon ligand binding. The nature of the cocaine aptamer makes it an ideal candidate for such an assembly. Interestingly, it has been shown that the cocaine aptamer binds quinine with an affinity that is 30- to 50-fold higher than cocaine (107,108). This property was used to design a model system able to trigger the assembly of the two fragments that will elongate to an aptachain (109). The concept was further implemented as a biosensor by immobilization of one fragment on AuNPs, which induced a shift of their plasmonic resonance and a visible color change upon quinine binding (LOD = 1.1 μM, Figure 3F).

A similar approach was used recently for target induced construction of a hand-in-hand RNA nanowire. In the absence of theophylline as the specific ligand, RNA split aptamer fragments were designed to be stable hairpins. When theophylline was present, hairpin opening of the RNA probe and subsequent assembly of an RNA nanowire was triggered. The nanowire was captured on an electrode interface and sensed with silver nanoparticles as electrochemical species (LOD = 50 nM) (110).

## SPLIT DNAzymes

### DNAzymes

**Figure 4.**
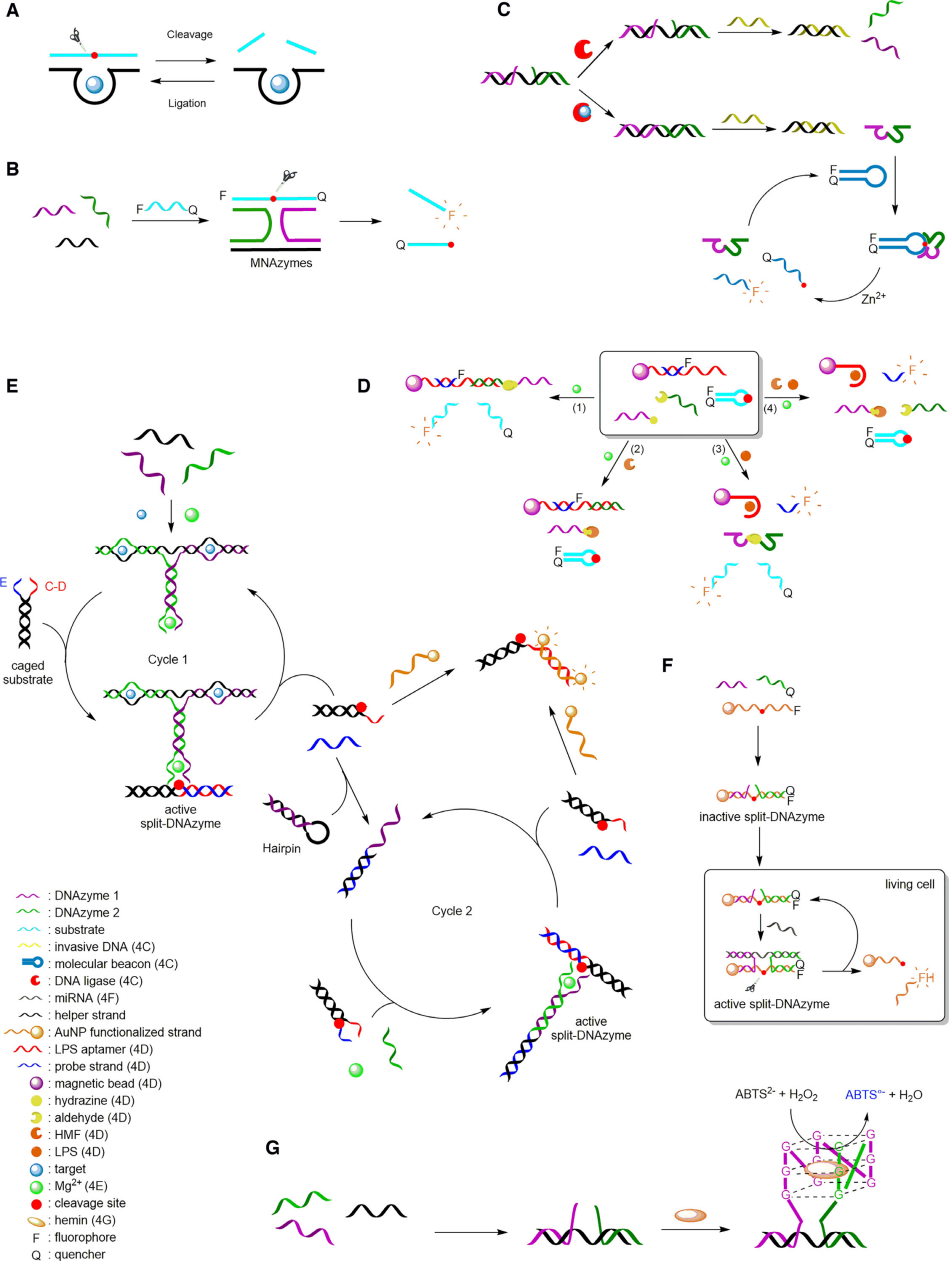
Target detection by split DNAzymes. (**A**) Catalytic activities of DNAzymes: cleavage and ligation reaction (111). (**B**) General scheme of a MNAzyme assay (121). (**C**) ATP and NAD^+^ detection by combining split 8–17 DNAzymes and CAMB (127). (**D**) HMF and LPS detection by hydrazone chemistry assisted DNAzyme (128). (**E**) Signal amplification for the detection of Hg^2+^ (129). (**F**) Detection of miRNA in living cells by a split DNAzyme and AuNPs (131). (**G**) Split peroxidase mimicking DNAzyme principle (133).

DNAzymes are DNA single-strands having catalytic activities owing to their three-dimensional structure stabilized by hydrogen bonding, π-stacking and metal-ion coordination. Obtained by *in vitro* selection methods, many deoxyribozymes have been evolved to catalyze RNA cleavage, RNA and DNA ligation, and a variety of covalent modification reactions of nucleic acid substrates (Figure 4A) (111). There are different types of DNAzymes, but the most prominent are ribonucleases, which catalyze the cleavage of a ribonucleotide phosphodiester bond by a transesterification reaction triggered by a metal ion (112). The first DNAzyme has been discovered in 1994 by Breaker and Joyce, and is a Pb^2+^-dependent RNA cleaving DNAzyme (6,113). Most of these DNAzymes are composed of a catalytic site flanked by two binding arms able to recognize the nucleic acid substrate. This also applies to the two well studied DNAzymes that are the **8–17** and the **10–23** DNAzyme (114). Another category of DNAzymes are Ligases, which join molecules instead of fragmenting them. The first example was described by Cuenoud and Szostak in 1995, in which the Zn^2+^- or Cu^2+^-dependent metalloenzyme catalyses the condensation of a 5′-hydroxyl with a 3′-phosphorimidazolide to form a new phosphodiester bond (115). The use of DNAzymes is continually progressing and the reaction scope of catalysis by DNA as well as their numerous applications in biochemistry have been recently reviewed by key players in the field (8,9,112,116–120).

### Split DNAzymes

Considering the advantages offered by split aptamers, split DNAzymes naturally have emerged as new functional biosensors. Composed of multiple oligonucleotides capable of assembling into the active DNAzyme core in the presence of a helper strand, these architectures were called MNAzymes for **M**ulticomponent **N**ucleic **A**cid en**zymes**, and proved their high efficiency for nucleic acid detection and mismatch discrimination (Figure 4B) (121–126).

With the purpose of detecting ATP or nicotinamide adenine dinucleotide (NAD^+^), Lu *et al.* developed a ligation-triggered DNAzyme cascade by combining a split DNAzyme with a catalytic and molecular beacon (CAMB) strategy (127). The **8–17**-DNAzyme was split into two fragments, which are inactive toward the molecular beacon strand in the absence of DNA ligase and the cofactor Zn^2+^ (Figure 4C). The addition of a complementary strand triggers the hybridization of both fragments to become ligated by ATP-dependent T4 DNA Ligase (for ATP detection) or NAD^+^-dependent *E. coli* DNA ligase (for NAD^+^ detection). The amount of newly formed DNAzyme was found being directly related to the concentration of the cofactor ATP or NAD^+^. The addition of an invasive DNA frees the **8–17**-DNAzyme, which subsequently is hybridized with a hairpin-structured molecular beacon substrate. Addition of the metal co-factor activates the DNAzyme and cleaves the molecular beacon substrate, thereby producing a significant increase of fluorescence. The LOD was found to be 100 pM for ATP and 50 pM for NAD^+^.

Very recently, the split **8–17** DNAzyme was also used for the detection of double targets, namely 5-hydroxymethylfurfural (HMF) and lipopolysaccharide (LPS) (128). The design involves a hydrazine group at the extremity of the first fragment, while the second fragment carries an aldehyde group. Under standard conditions, the two fragments react to form the corresponding DNAzyme modified by a hydrazone linkage (Figure 4D). In the presence of Mg^2+^ ions and a molecular beacon substrate strand labelled with a fluorescent probe and a quencher, a strong fluorescence emission is observed, thus indicating that the hydrazone linkage does not significantly influence the catalytic activity of the DNAzyme (Figure 4D1). In the presence of HMF only, the aldehyde group present in the structure inhibits the formation of the DNAzyme, and hence the cleavage of the substrate, leading to no fluorescence emission (Figure 4D2). The presence of LPS is visualized by magnetic beads functionalized with an LPS aptamer and a fluorescent probe strand hybridized to the aptamer, such that fluorescence is quenched. A conformational change induced by the binding of LPS to its aptamer induces the release of the probe strand and thus fluorescence emission. Therefore, in the presence of LPS only, fluorescence of both the DNAzyme cleaved substrate and the probe strand released from the aptamer is observed (Figure 4D3). In the presence of both, HMF and LPS, formation of the DNAzyme and consequently cleavage of the molecular beacon substrate is inhibited, such that merely fluorescence of the probe strand is observed (Figure 4D4). The limits of detection have been evaluated to be 0.04 μM for HMF in a concentration range from 0.01 μM to 5 μM, and 0.08 ng/mL for LPS in a concentration range from 0.08 to 2000 ng/ml.

Split DNAzyme fragments have also been used to engineer an ultrasensitive biosensor for Hg^2+^ detection (129). Many techniques mainly based on colorimetry or fluorescence have been previously developed to detect this divalent metal ion, achieving high selectivity, but only moderate sensitivity (130). The DNAzyme based biosensor combines a Mg^2+^-dependent split DNAzyme (two fragments), a binding DNA, a hairpin DNA and a caged substrate, and is also based on colorimetry (Figure 4E). The presence of Hg^2+^ is monitored through a cascade signal amplification. In the presence of Hg^2+^, the DNAzyme fragments bind a third DNA strand (helper strand) supported by the formation of T–Hg^2+^–T base pairs, thus resulting in the formation of an active DNAzyme able to cleave the caged substrate and release segments E and C–D. The C–D segment will then bind to AuNPs forming an aggregate network that becomes blue, allowing detection of Hg^2+^ by the naked eye. Segment E can initiate another signal amplification process by opening the hairpin containing one part of the split DNAzyme leading to signal amplification with a LOD value of 10 pM. Furthermore, split DNAzymes have been used to sense miRNA in living cells. The system relies on AuNPs functionalized with a 3′-FAM-labeled substrate strand hybridized to the two fragments derived from the Mg^2+^-dependent **10–23** DNAzyme, one of them carrying a quencher at its 5′-end (Figure 4F) (131). In the absence of the target miRNA, the split DNAzyme is inactive and the fluorophore is quenched by both the quencher and the AuNP. However, the presence of the target miRNA promotes the formation of an active split DNAzyme that cleaves the substrate and releases the fluorophore from the quencher. Simultaneously, the release of the target promotes another cycle of activation, thus allowing an amplification of the fluorescent signal and a LOD of the target miRNA in living cells of 10 pM.

### Split peroxidase mimicking DNAzymes

The peroxidase mimicking DNAzyme, initially designed by Travascio *et al.* in 1998 (132) and then split for the first time by Deng *et al.* in 2008 (133), is actually a hemin-binding DNA aptamer, which exhibits peroxidase activity. The DNAzyme sequence possesses four GGG motifs and is thus able to self-assemble into a G-quadruplex structure, which can catalyze the H_2_O_2_-based oxidation of 2,2′-azido-bis(3-ethylbenzothiozoline-6-sulfonic acid) (ABTS^2−^). The first split DNAzyme was divided into two fragments in a 3:1 split mode (133) (Figure 4G), and many studies have been devoted to reveal the best architectural features and reaction conditions to achieve highly effective detection of nucleic acid sequences (134), mismatches (135), carcinoembryonic antigen (136), enzymes (137,138), metal ions (139–141), small molecules (142,143) or drugs (144–146).

Compared to the vast number of existing aptamers it is obvious that only a small number of them have been successfully translated into split systems. Splitting designs require a judicious choice of the fragments that do not disturb the stability and the affinity of the binding site. This is especially true with aptamers having hairpin and three-way junction architectures (86). While most systems are based on fragments that would reassemble non-covalently upon binding of the ligand, split aptamer covalent ligation strategies might overcome the difficulties encountered with non-covalent hairpin and three-way junction architectures and are currently emerging as an efficient alternative for the detection of small molecules (100,128). However, given the versatile applications of DNA split aptamers, the engineering and characterization of new architectures as biosensors are rapidly expanding (147). Recent examples include the use of different split fragments for the detection of tumor cells (148–150), viruses (151,152), antibiotics (153), antigens (154,155), exosomes (156) or lyzozyme in the residues of latent fingerprints (157).

## SPLIT APTAZYMES

There are a few examples of combination of a DNAzyme/ribozyme with an aptamer in a split design, hence called split aptazyme or short, splitzyme, to be applied as biosensor. In general, activity of an aptazyme is dependent on the presence of a specific ligand that binds to the aptamer region.

As a prominent example, an RNA-branching deoxyribozyme was engineered as a splitzyme to respond positively to ATP, resulting in modulated control of its activity (158). For this purpose, an in vitro selected DNAzyme that supports reaction of the 5′-phosphate of one RNA strand with an internal 2′-OH group of another RNA strand, was reengineered to contain the ATP aptamer in its structure. To form the splitzyme, the stem-loop of the aptamer was replaced by three base pairs, and a break was introduced in the phosphodiester backbone, thereby requiring two oligonucleotides to form the complete deoxyribozyme. An optimized version of the splitzyme reached a high ligation yield (∼75–80%) in the presence of millimolar concentrations of ATP, although only after 22 h. In another approach, a split version of an aptazyme was combined with molecular beacons that act as substrate and contain a ribonucleotide at the cleavage site (159). The aptazyme, composed of the **8–17** DNAzyme and the adenosine responsive aptamer, was split into two fragments. Similar to the set-up depicted in Figure 4C (127), a molecular beacon substrate labelled with a fluorescent dye and a quencher, was used to monitor split aptazyme activity. In the absence of adenosine, the split aptazyme is inactive, thus leaving the MS intact and resulting in low fluorescence. When adenosine was added to the medium, the two aptazyme fragments interact to form the catalytically competent structure and cleave the molecular beacon substrate, which results in significant increase of the fluorescence signal. The LOD for this system was found to be 1 μM (159), and thus two orders of magnitude lower than observed with a previous split aptazyme-based adenosine sensor (160). Also an RNA enzyme, the hammerhead ribozyme (HHR) has been used in combination with an aptamer in a split aptazyme design (161) as will be described in more detail below (section Split Spinach).

## GFP-MIMICKING LIGHT UP RNA APTAMERS

**Figure 5.**
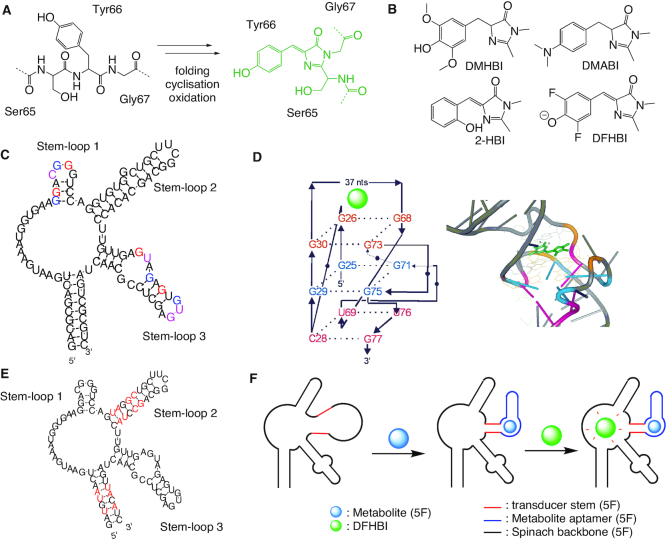
(**A**) The three amino acid residues Ser65-Tyr66-Gly67 in the center of GFP, forming the chromophore by intramolecular cyclization and oxidation (165). (**B**) GFP-like fluorophore structures (175). (**C**) Secondary structure of the Spinach aptamer (175). The colored bases refer to the colors used in the G-quadruplex shown in (**D**). (**D**) Cartoon presentation (left) and 3D structure (right) of the G-quadruplex interacting with DFHBI (PDB code: 4TS2) (176). (**E**) Secondary structure of the Spinach 2 ‘superfolder’. Red bases are the mutations as compared with the parent Spinach aptamer in (**C**) (177). (**F**) Structure of the Spinach sensor ligated to a metabolite aptamer via a transducer stem (178).

Over the past decades, it has been shown that RNA plays crucial roles in the living cell and moreover, can be engineered into a tool with numerous potential applications. To elucidate the many functions of RNA and to create new drugs or models of gene expression networks, knowledge of RNA localization, dynamics, and regulation is important. Therefore, many groups developed methods for fluorescence imaging and detection of DNA and RNA in living cells in real-time (162,163). The main problem of RNA imaging is that RNA cannot be easily visualized, and thus has to be tagged with a fluorescent probe, which however is challenging in living cells. A solution to this problem are light-up RNA aptamers that mimic the green fluorescent proteins (GFPs). Since 1990, GFPs have been used to detect proteins in the cellular environment. The first GFP was discovered by Shimomura *et al.* from the jellyfish *Aequorea Victoria* (164). The 4-hydroxybenzylidene imidazolinone (HBI), which is formed from intramolecular reaction of the three amino acid residues Ser65–Tyr66–Gly67, is responsible for the fluorescence in GFP (Figure 5A). To form the chromophore, the residues are placed in the β-barrel of the protein to proceed through a cyclization and oxidation step (165). Therefore, the chromophore is localized in the center of the protein and is protected from the environment to activate the fluorescence. Thus, GFPs are advantageous as compared to other proteins that bind their chromophores *via* non-covalent interactions and thus are dependent on their environment.

Taking the working mode of GFPs as a lead, light-up aptamers have been developed by in vitro selection (166–171). Those aptamers create fluorescence upon binding their specific ligands *via* non-covalent interactions, and hence do not possess the advantage of GFPs that is harboring the chromophore as an integral part of their structure. Yet, light-up aptamers are seen as GFP mimics based on the fact, that fluorescence of, in the unbound state non-fluorescent, ligands is created by embedding those in a suitable environment. Thus, light-up aptamers are considered being label-free fluorescence probes. The ligand binds tightly to the nucleic acid, i.e. by intercalating or as minor groove binder. As a result, intramolecular movements are limited and, upon excitation, radiative relaxation can occur, resulting in an increase of fluorescence. Light-up aptamers can be fused to RNAs of interest allowing their visualization by optical methods (167,170). Furthermore, light-up aptamers have been engineered into aptasensors for detection of pathogens in food or water samples (172). The Malachite green aptamer (MGA) was the first RNA mimic of GFP to show a fluorescence response upon binding to malachite green (MG), a triphenylmethane dye with *K*_D_ ≤ 1 μM, and splitting MG aptamers allowed for fluorescence detection of nucleic acids (173,174). However, Malachite Green and its derivatives are highly cytotoxic, and therefore, other dyes were needed to develop methods for nucleic acid detection in living cells.

## SPINACH RNA APTAMER

### Properties

In an effort to develop non-toxic light-up aptamers, various GFP-like fluorophores like 3,5-dimethoxy-4-hydroxybenzylidene imidazolinone (DMHBI), 4-dimethylamino-benzylidene imidazolinone (DMABI), 2-hydroxybenzylidene imidazolinone (2-HBI) and 3,5-difluoro-4-hydroxybenzylidene imidazolinone (DFHBI) (Figure 5B) were synthesized and used for *in vitro* selection (175). The brightest fluorophore among those was DFHBI and, because of the green fluorescence of the aptamer bound dye, the system was named like the green vegetable Spinach. As compared to the other HBI derivatives, DFHBI exhibits specific fluorescence, also in the cellular environment, and does not induce cytotoxicity. The sequence is formed of 98 nucleotides, folded in three stem-loops (Figure 5C). In the three-dimensional fold, the three tetrade quadruplex is stabilized by two K^+^ ions (176). DFHBI binds on the top of the G-quadruplex motif to activate the fluorescence (Figure 5D). Different interactions are involved. Residue G30 binds to the DFHBI carbonyl oxygen and interacts with the benzylidene carbon *via* Van der Waals interactions, N3 of DFHBI binds to the 2′-OH of A64, and the 2′-OH of G26 binds to the phenolate oxygen of DFHBI. One of the two K^+^ ions coordinates two of the three G-quartets, and the other K^+^ ion the third lower G-quartet. The understanding of the Spinach structure allowed the minimization of the aptamer to generate a ‘Baby Spinach’ system, 51 nucleotides long, without changed fluorescence properties when binding to DFHBI (175).

The brightness of the Spinach-DFHBI system is only 53% of that of EGFP or 80% of the GFP brightness, respectively. In addition, Spinach turned out to misfold in cells (175,177). Thus, the fusion of Spinach with an RNA of interest remained challenging and showed rather low fluorescence. Performance of Spinach was improved by a number of sequence changes to stabilize the aptamer fold and to enhance the fluorescence in *in vivo* systems. Thus, Spinach 2, a ‘superfolder’ aptamer showing enhanced fluorescence upon fluorophore binding was developed (Figure 5E) (177). Compared to Spinach, Spinach 2 has almost the same photophysical properties, the excitation/emission spectra are similar, but show higher brightness. Owing to the improved folding in living cells, the Spinach 2 aptamer allowed to image toxic CGG-repeat-containing RNAs and retained 80% of its fluorescence, while Spinach did not show any fluorescence in an identical set-up.

Spinach systems have been used for the detection of small molecules (178) and specific nucleic acid sequences (179), as well as for imaging protein expression (180) and RNA transcription (181). To acquire a broad range of applications and to image metabolites like for example ADP, a sensor has been developed composed of the Spinach aptamer and a second aptamer that binds to small molecules (178). Therein, stem-loop 2 (see Figure 5E) is essential for the stabilization and the activation of the Spinach fluorescence. This stem-loop has been replaced by a transducer stem connecting the Spinach backbone with the chosen metabolite aptamer. The transducer is stabilized only when the target molecule is bound to the aptamer, which in turn allows proper folding of the Spinach aptamer and fluorescence emission when bound to DFHBI (Figure 5F).

### Split Spinach

**Figure 6.**
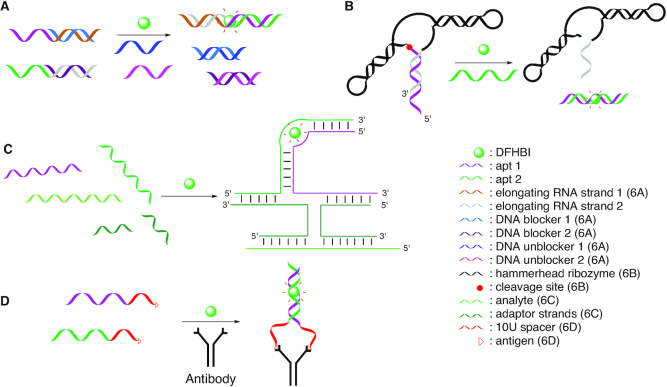
(**A**) Monitoring split Spinach assembly (182). (**B**) Hammerhead ribozyme cleavage assay using the split Spinach aptamer (161). (**C**) Universal Split Spinach aptamer assay (183). (**D**) Split Spinach aptamer assay for antibody detection (185).

The use of light up aptamers in split scenarios has potential in particular for the visualization of RNA assembly. To this end, Spinach was cut in two fragments that, upon appropriate interaction, would form the full length aptamer (182). In order to find a suitable split region, structure-function relationship studies have been carried out. Stem-loops 1 and 3 are responsible for the formation of the G-quadruplex structure, which is essential for binding of DHFBI (Figure 5C–E). Therefore, stem–loop 2 of the Spinach system provides an excellent region for sequence modifications and is a good candidate for the split region (176,178,182). First studies of the split Spinach assembly involved DNA blocker sequences to temporarily control the assembly of the functional aptamer. The split site is located within the stabilizing stem–loop 2, and the two fragments are elongated. Two DNA strands (DNA blockers 1 and 2 in Figure 6A) are designed to form duplexes with each of the two Spinach fragments to prevent folding into the functional aptamer. Association of the two pieces is permitted, when two complementary ‘unblocking’ DNA strands are added, allowing formation of the DFHBI binding pocket, mirrored in DFHBI fluorescence (Figure 6A) (182).

Split systems can also be utilized for RNA self-cleavage detection and quantification. Thus, the hammerhead ribozyme (HHR) has been combined with one of the Spinach aptamer fragments (Figure 6B). The Spinach aptamer used in this study has been minimized, and one fragment (apt 1 in Figure 6B) is used to form the 5′-part of the hammerhead substrate sequence. A short sequence complementary to this Spinach fragment is added to the 3′-end of the HHR, thus forming a short stem structure in the functional ribozyme. Upon self-cleavage, the elongated ribozyme strand is easily displaced from the Spinach fragment by addition of the second aptamer fragment (apt 2 in Figure 6B), thus allowing formation of the functional Spinach aptamer, mirrored in high fluorescence in the presence of DFHBI (Figure 6B) (161). This method is easily adaptable and can be used to analyze the performance of different ribozyme variants.

As seen with the HHR, application of the Spinach aptamer for detection of activity requires sequence adaptation, hence making necessary all the different steps of setting up such a system, like design, synthesis, purification, and optimization. In 2017, Kikuchi *et al.* developed a Universal Split Spinach Aptamer (USSA) probe able to bind DNA or RNA targets independent of their sequences (183). In the process, the Spinach fragments (apt 1 and apt 2; Figure 6C) have to interact with two adaptor strands, which only upon hybridization to the target would re-form the binding site for DFHBI. In this way, split aptamers are not in direct contact with the target and hence can be used in a universal manner for sensing different RNA or DNA targets (Figure 6C) with a LOD of 1.5 nM in the best case. However, the detection of mRNAs using the USSA still suffered from low-brightness, owing to thermal instability as well as K^+^ and Mg^2+^ concentration-dependent folding, and thus remained challenging. Wang *et al.* improved the strategy and developed an aptamer-initiated fluorescence complementation protocol for RNA imaging, based on two fragments of Baby Spinach that are functionalized with complementary sequences to the mRNA target. When the mRNA target is present, both fragments become oriented sufficiently close to one another for formation of the functional aptamer, efficient DFHBI binding and high fluorescence (184).

Many of the processes in living systems take place due to the proximity of the components. Thus, split Spinach aptamers have been used not only with nucleic acids, but also for detection of a specific antibody *via* the corresponding antigens conjugated at the end of both Spinach fragments (Figure 6D). The functionality of the split strands is retained by an additional spacer, comprising 10 uracil units, between the split Spinach sequences and the antigen. In the presence of the specific antibody, the fragments are sufficiently close to properly fold and to bind the DFHBI fluorophore, thus resulting in a fluorescence increase (185).

## BROCCOLI RNA APTAMER

### Broccoli - a new green light up aptamer

The Spinach aptamer was obtained from in vitro selection by the SELEX method. This however, is based on the affinity of the aptamer for the target/fluorophore and not on its capability of activating the fluorescence of the fluorophore (175). Thus, as mentioned above, Spinach turned out being not optimal for *in vivo* experiments (186). To overcome this problem and to improve RNA imaging by light-up aptamers, research efforts were started to develop aptamers with similar features like Spinach, but improved characteristics for application in living cells. Filonov *et al.* invented a new selection method combining the classic SELEX procedure in *E. coli* with a fluorescence-activated cell sorting step, from which a new light-up aptamer, named Broccoli was obtained (187). With a sequence length of 49 nucleotides, Broccoli is shorter than Spinach, and exhibits better folding efficiency under physiological conditions, green fluorescence upon binding DFHBI, and lower magnesium ions dependence.

### Split Broccoli

**Figure 7.**
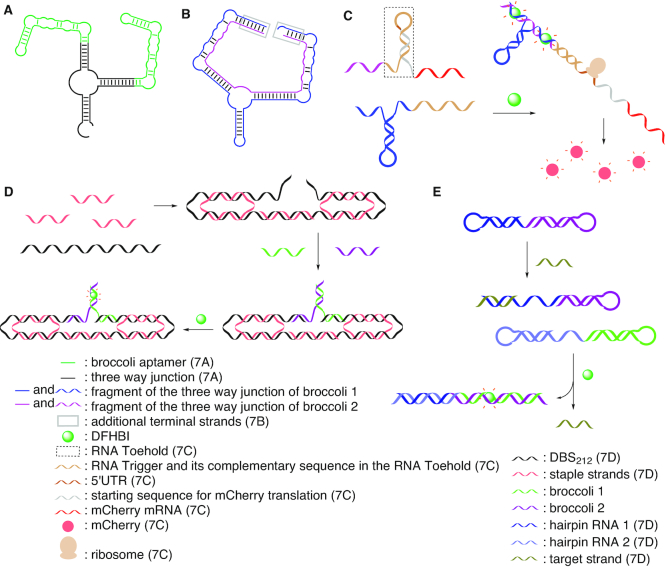
(**A**) The three-way junction of the monomeric Broccoli aptamer (190). (**B**) The dimeric three-way junction Broccoli aptamer (190). (**C**) Formation of the dimeric Broccoli aptamer for monitoring RNA–RNA hybridization events (190). (**D**) Monitoring the folding of an RNA origami (191). (**E**) CHARGE system for detection of specific nucleic acid targets (192).

Because of its favorable properties, the Broccoli aptamer has also been studied as split variants (188,189). For this purpose, two Broccoli aptamers were combined in a three-way junction structure (Figure 7A), and this structure was subsequently re-designed to consist of two separate RNA-strands with the split sites being located in the terminal loops of the two Broccoli hairpins (Figure 7B) (190).

Additional terminal strands were added to stabilize the system against nuclease degradation. This system was used to monitor RNA–RNA hybridization events in vivo (190), as well as for in cell mRNA imaging (184). For this, one three-way junction forming Broccoli fragment (Broccoli 1) was functionalized with an RNA ‘Trigger’ sequence, and another three-way junction forming Broccoli fragment (Broccoli 2) with an RNA ‘Toehold’ and the mCherry mRNA. The Trigger and the Toehold have complementary sequences allowing control of mCherry expression. The RNA Toehold forms a hairpin structure containing the 5′-UTR of mCherrry. Upon hybridization, the two three-way junction forming Broccoli fragments self-assemble into the functional system, and allow for emission of green fluorescence in the presence of DFHBI. In addition, the sequence of the Toehold comprising the 5′-UTR is set free to favor ribosome binding and initiation of translation to express mCherry (Figure 7C). The green fluorescence of the split aptamer confirms the RNA-RNA hybridization event, and the red fluorescence of the mCherry protein indicates successful activation of translation.

Light-up aptamers are also valuable tools for verification of proper folding of DNA and RNA nanostructures. In this context a split Broccoli aptamer system has recently been used to monitor the folding of an RNA origami (Figure 7D) (191). An RNA origami ribbon was formed from the 212 nucleotides long De Bruijn sequence (DBS) scaffold, designed to adopt a closed conformation only in the presence of the split Broccoli scaffold, and folding was induced by addition of specific staple strands. The Broccoli aptamer is split at the terminal stem loop; the two resulting Broccoli fragments are extended via an eight nucleotides spacer with complementary sequences, allowing specific interaction with the DBS scaffold. Formation of the ribbon was followed by observing the fluorescence of the added DFHBI-1T dye.

Furthermore, based on a Toehold-mediated strand displacement mechanism, a novel **C**atalytic **H**airpin **A**ssembly **R**NA circuit sensor that is **G**enetically **E**ncoded (named CHARGE) was designed to detect specific RNA molecules inside living cells with high sensitivity (192). The two ends of complementary hairpin RNA strands were functionalized with two non-fluorescent fragments of the Broccoli aptamer (Figure 7E). The target strand to be detected serves the role of invading one of the hairpin structures containing one Broccoli fragment, thus opening up the stem loop and inducing hybridization with the other hairpin structure containing the second Broccoli fragment. Thus, the functional Broccoli aptamer is formed as monitored by the emerging fluorescence upon addition of the cognate dye DFHBI-1T. Target concentrations as low as 0.5 nM could be detected.

### Other split RNA aptamers

Apart from light-up aptamers, other RNA aptamers have been used in split designs for specific target detection. A split version of an aptamer specific for the RNA-binding protein eIF4A was used in combination with the protein complementation approach to detect and localize specific mRNAs in live *E. coli* cells (193). The assay relies on the sequence specific binding of two RNA probes complementary to two adjacent sites on the mRNA target. Each RNA probe is composed of a sequence complementary to the target and a fragment of the split aptamer, connected by a flexible linker. In the presence of the target mRNA, binding of the two RNA probes next to one another, brings the two aptamer fragments in close proximity and hence supports assembly. The assembled aptamer binds to a split version of its target protein eIF4A, and thereby triggers re-association. elF4A was used here as a fusion protein with EGFP, hence the emerging fluorescence upon aptamer and protein assembly was used as readout. Compared with assays where the full size EGFP and/or full length aptamer was used, the combination of the split aptamer approach with protein complementation lead to significant fluorescence background reduction and allowed to detect, in live cells, a signal from a specific mRNA with an average RNA concentration of ≤1 molecule per cell.

Another example of a non-light up split aptamer assay is detection of theophylline by aptamer triggered colorimetric aggregation of gold nanoparticles (AuNPs) (194). The theophylline RNA aptamer was cut into two fragments. The fragments interact with the AuNPs, enhancing the salt tolerance of the nanoparticles and preventing aggregation. Addition of theophylline triggered the assembly of the RNA aptamer fragments, thus reducing the amount of RNA available to protect the AuNPs and allowing their salt-induced aggregation. Aggregation is associated with a color change from red to blue, that can be visually detected or measured by absorption spectroscopy. The relatively low LOD (67 nM of theophylline), the high selectivity and the facile visual detection make this assay a good tool for diagnostic applications and easily adaptable to other RNA aptamers.

## SPLIT RIBOZYMES

As for split studies on RNA aptamers, the strategy of fragmented systems has been used with ribozymes in order to improve *in vivo* detection methods. Ribozymes are RNA molecules that can catalyze a wide range of reactions, although ribozymes occurring in nature are limited to catalysis of *trans*-esterification, hydrolytic or peptidyl transfer reactions (195). Nowadays, the mechanisms and structures of catalytic RNAs have been studied to an extent that has allowed to create tools for application in molecular biology, medicine or analytics.

### Split hairpin ribozyme

Basically driven by the technical challenges of chemically synthesizing long RNA strands, ribozymes have been assembled from two or more fragments in mechanism and structure studies. For example, with the hairpin ribozyme, a small naturally occurring catalytic RNA derived from the (–)-strand of the *Tobacco Ringspot* virus satellite RNA (196), the terminal hairpin loop closing one of the helices has been removed, thus splitting the ribozyme strand into two parts. In order to compensate the stability loss, the helix was extended by three additional Watson–Crick base pairs. Both ribozyme versions have virtually the same cleavage characteristics, and thus both set-ups have been extensively used in numerous studies (197).

### Split group I intron ribozyme

The group I intron of the ciliated protozoan *Tetrahymena thermophila* is an RNA catalyst that supports mRNA self-splicing (198). A split version of the *Tetrahymena* ribozyme was engineered to develop an mRNA detection method in mammalian cells (199). The ribozyme fragments were connected at one end to mRNA antisense strands *via* linker sequences. On the other end, the ribozyme fragments were attached each to one half of the coding sequence of nonsecreted TEM-1 β-lactamase. In the presence of the mRNA target, the antisense strands would bind to it, thus positioning the ribozyme fragments for functional assembly. Upon ribozyme mediated splicing, the complete coding sequence of the TEM-1 β-lactamase was regained for translation to occur. The synthesized proteins were then detected by a fluorogenic substrate, that is β-lactam linked to an umbelliferone called CC1. The produced β-lactamase catalyzed the hydrolysis of CC1, thus releasing umbelliferone and inducing a fluorescent signal (200). The key feature of this system is its inherent capability of signal amplification, since one molecule of target RNA induces the production of many protein molecules from the spliced RNA. The assay is universal, since different mRNA targets can be detected by adapting the sequence of the antisense strands to the target of interest.

## LIMITATIONS AND FUTURE PERSPECTIVES

Despite the high potential of split aptamers for applications in diagnostic and therapy, only a rather small number of split structures have been successfully used so far. This is certainly a consequence of the fact that the design of a well-performing split system is still a challenging task. Basically there are three key issues that require careful consideration: (i) choice of the split site, (ii) stability of the split aptamer/nucleic acid enzyme target complex and (iii) sensitivity of the split system.

### Choice of the split site

When designing split systems, it has to be taken into account that the three-dimensional structure of the parent nucleic acid strand can be considerably perturbed, if split at an unfavorable position. Therefore, careful design of the fragments and verification of the assembled structure by theoretical (computer aided) and experimental analysis is a very important task. This requires the understanding of the aptamer/nucleic acid enzyme target complex including the role of individual functional groups of nucleobases and of the sugar-phosphate backbone. In order to maintain the functionality of the system, splitting is usually restricted to functionally dispensable sites. Such sites are usually available in DNAzymes and ribozymes, in aptamers however, they are often missing. Recent work by Wang *et al.* has addressed this problem by studying split aptamers with broken initial small molecule binding pockets, and demonstrating, in wet lab experiments as well as in silico, that biorecognition and binding capabilities are preserved (201). Thus, the prediction of ligand binding potency of split aptamer designs by MD simulations can be expected to enhance the future development of split aptamers for biosensing purposes.

### Stability of the split aptamer/nucleic acid enzyme–target complex

Compared to the parent full-length nucleic acid strand, the individual fragments of a split system often have lower affinity to the target, which results in reduced stability of the aptamer/nucleic acid enzyme target complex. Furthermore, there is a higher entropic cost of complex formation, due to the fact that two or more fragments need to assemble instead of only one nucleic acid strand that binds to the target. Stabilization of the split system can be achieved by structural manipulation, *i.e*. extension of the individual fragments by additional nucleotides that by complementary base pairing would assist assembly and increase stability of the split aptamer/nucleic acid enzyme target complex. Instead of additional nucleotides also other functionalities that support target-induced fusion of the fragments can be used. A prominent example is the work by Jin *et al.* (69) described above, who attached pyrene moieties at the ends of two split aptamer fragments, such that formation of the pyrene dimer upon split aptamer folding in the presence of the cognate ligand not only allowed for detection, but in addition assisted assembly and increased stability of the aptamer target complex. In another scenario, the fragments are functionalized with reactive groups, as for example azides and alkynes or aldehydes and amines/hydrazines, such that the individual strands, upon folding, would become covalently linked by an appropriate chemistry (100,102,103). In this way, upon target induced assembly of the split system, the effective molarity of the reactive groups attached to the ends of the split fragments would be significantly increased, thus promoting chemical reaction. This in turn would stabilize the split aptamer/nucleic acid enzyme target complex, and moreover can be used as readout of target-induced assembly and thus for detection, and potentially sensing, of the cognate ligand.

### Sensitivity of the split system

The problem of limited affinity and stability of split aptamer/nucleic acid enzyme target complexes is also mirrored in reduced sensitivity. Although impressive sensitivity has been demonstrated in some cases (Table 1), there is a particular need for signal amplification strategies to improve the sensitivity down to LOD in the picomolar range. This is challenging with split systems and usually requires rather complex assays. In a quite straight-forward way signal amplification is achieved by combination of the split system with protein enzymes to turnover a specific substrate, which is available to the enzyme only if the split aptamer target complex has formed (202). In an analogous scenario, aptazymes cleave or ligate specific substrates only in the presence of a specific ligand that binds to the aptamer region of the aptazyme and stabilizes the functionally competent conformation of the catalytic core. Other methodologies like for example strand displacement amplification of a sequence that becomes available exclusively upon formation of the split aptamer target complex and detection/sensing of the formed dsDNA in a real time PCR assay (203) have started to be integrated in split aptamer and nucleic acid enzyme designs.

**Table 1.** Summary of split-systems described in this review. LOD: limit of detection; FAM: 6-carboxy-fluorescein; FRET: Förster resonance energy transfer; ATP: Adenosine triphosphate; BHQ: black hole quencher; Cy3/Cy5: Cyanines 3 and 5; SPR: surface plasmon resonance; ALO: aryl-less cyclooctyne; DIBAC: dibenzoazacyclooctyne; NAD: nicotinamide adenine dinucleotide; CAMB: catalytic and molecular beacon; HMF: 5-hydroxymethylfurfural; LPS: lipopolysaccharide

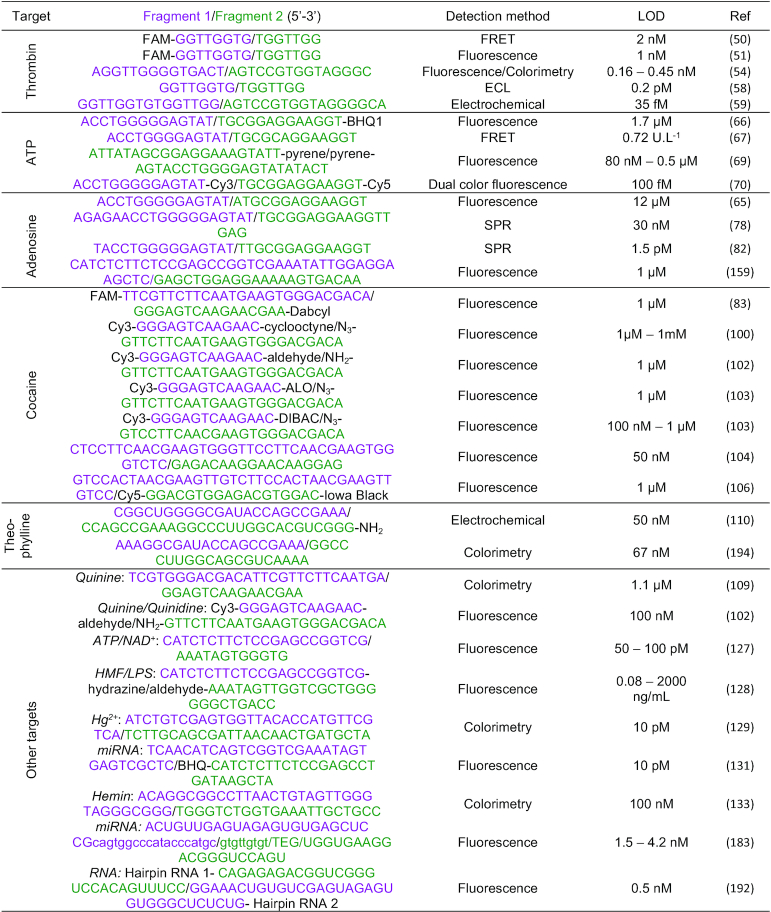

## CONCLUSION

Split aptamers and ribozymes/DNAzymes are at the initial stage of design and application. The concept of split aptamers/nucleic acid enzymes involves dissecting the parent full length strand in a series of two or more independent and non-functional fragments, which in the presence of the specific target would assemble to form the functional entity. Thus, in comparison to full length functional nucleic acids, split systems are easier to synthesize, and due to their smaller size are less prone to form unwanted secondary structures, which might deliver false-positive or non-specific signals in the absence of the target. The ideal design would include that the fragments of the split system are brought in close contact and deliver a signal only when the target is present. Aptamers are the key elements for detecting small molecules or proteins, whereas nucleic acid enzymes have been applied mainly for detection of specific nucleic acid sequences or metal ions. The combination of both, aptamers and nucleic acid enzymes, into aptazymes is an appealing concept of signal enhancement by the multiple turnover of the enzyme that amplifies the binding event of the target to the aptamer region into multiple reaction products used for readout.

Overall, the design and application of split aptamers and nucleic acid enzymes in biosensing applications is still in its infancy and remains challenging. Further improving the stability of split nucleic acid target complexes and with that the sensitivity is an important task. In addition, fast and practical approaches based on fluorescence, chemiluminescence, colorimetric or electrochemical detection need to be further implemented in split aptamer/nucleic acid enzyme assays to achieve efficient working modes. Nevertheless, the so far reported functional assemblies derived from aptamers and ribozymes/DNAzymes, impressively demonstrate the proof of principle, and pave the way for deeper implementations and exciting new developments in this compelling and emerging field.

## References

[1] Li T., Dong S., Wang E. G-quadruplex aptamers with peroxidase-like DNAzyme functions: which is the best and how does it work. Chem. – Asian J. 2009; 4:918–922. 19422006 10.1002/asia.200900019

[2] Neo J.L., Kamaladasan K., Uttamchandani M. G-Quadruplex based probes for visual detection and sensing. Curr. Pharm. Des. 2012; 18:2048–2057. 22380516 10.2174/138161212799958341

[3] Kosman J., Juskowiak B. Peroxidase-mimicking DNAzymes for biosensing applications: a review. Anal. Chim. Acta. 2011; 707:7–17. 22027115 10.1016/j.aca.2011.08.050

[4] Guerrier-Takada C., Gardiner K., Marsh T., Pace N., Altman S. The RNA moiety of ribonuclease P is the catalytic subunit of the enzyme. Cell. 1983; 35:849–857. 6197186 10.1016/0092-8674(83)90117-4

[5] Kruger K., Grabowski P.J., Zaug A.J., Sands J., Gottschling D.E., Cech T.R. Self-splicing RNA: autoexcision and autocyclization of the ribosomal RNA intervening sequence of tetrahymena. Cell. 1982; 31:147–157. 6297745 10.1016/0092-8674(82)90414-7

[6] Breaker R.R., Joyce G.F. A DNA enzyme that cleaves RNA. Chem. Biol. 1994; 1:223–229. 9383394 10.1016/1074-5521(94)90014-0

[7] Willner I., Shlyahovsky B., Zayats M., Willner B. DNAzymes for sensing, nanobiotechnology and logic gate applications. Chem. Soc. Rev. 2008; 37:1153–1165. 18497928 10.1039/b718428j

[8] Zhou W., Ding J., Liu J. Theranostic DNAzymes. Theranostics. 2017; 7:1010–1025. 28382172 10.7150/thno.17736 PMC5381262

[9] Peng H., Newbigging A.M., Wang Z., Tao J., Deng W., Le X.C., Zhang H. DNAzyme-mediated assays for amplified detection of nucleic acids and proteins. Anal. Chem. 2018; 90:190–207. 29183114 10.1021/acs.analchem.7b04926

[10] Ma L., Liu J. Catalytic nucleic acids: biochemistry, chemical biology, biosensors, and nanotechnology. iScience. 2020; 23:100815. 31954323 10.1016/j.isci.2019.100815 PMC6962706

[11] Parashar A. Aptamers in Therapeutics. J. Clin. Diagn. Res. 2016; 10:BE01–BE06. 10.7860/JCDR/2016/18712.7922 PMC4963637 27504277

[12] Klussmann S. Klussmann S The aptamer handbook: functional oligonucleotides and their applications. The Aptamer Handbook: Functional Oligonucleotides and Their Applications. 2006; Weinheim. Germany WILEY-VCH 490.

[13] Tuerk C., Gold L. Systematic evolution of ligands by exponential enrichment: RNA ligands to bacteriophage T4 DNA polymerase. Science. 1990; 249:505–510. 2200121 10.1126/science.2200121

[14] Gold L., Janjic N., Jarvis T., Schneider D., Walker J.J., Wilcox S.K., Zichi D. Aptamers and the RNA world, past and present. Cold Spring Harb. Perspect. Biol. 2012; 4:a003582. 21441582 10.1101/cshperspect.a003582 PMC3282410

[15] Sefah K., Shangguan D., Xiong X., O’Donoghue M.B., Tan W. Development of DNA aptamers using Cell-SELEX. Nat. Protoc. 2010; 5:1169–1185. 20539292 10.1038/nprot.2010.66

[16] Ellington A.D., Szostak J.W. In vitro selection of RNA molecules that bind specific ligands. Nature. 1990; 346:818–822. 1697402 10.1038/346818a0

[17] Bock L.C., Griffin L.C., Latham J.A., Vermaas E.H., Toole J.J. Selection of single-stranded DNA molecules that bind and inhibit human thrombin. Nature. 1992; 355:564–566. 1741036 10.1038/355564a0

[18] Kent A.D., Spiropulos N.G., Heemstra J.M. General approach for engineering small-molecule-binding DNA split aptamers. Anal. Chem. 2013; 85:9916–9923. 24033257 10.1021/ac402500n

[19] Hermann T., Patel D.J. Adaptive recognition by nucleic acid aptamers. Science. 2000; 287:820–825. 10657289 10.1126/science.287.5454.820

[20] Mayer G. The chemical biology of aptamers. Angew. Chem. Int. Ed. 2009; 48:2672–2689. 10.1002/anie.200804643 19319884

[21] O’Sullivan C.K. Aptasensors – the future of biosensing. Anal. Bioanal. Chem. 2002; 372:44–48. 11939212 10.1007/s00216-001-1189-3

[22] Xu F., Zhu Y.-C., Ma Z.-Y., Zhao W.-W., Xu J.-J., Chen H.-Y. An ultrasensitive energy-transfer based photoelectrochemical protein biosensor. Chem. Commun. 2016; 52:3034–3037. 10.1039/c5cc09963c 26790604

[23] Rimmele M. Nucleic acid aptamers as tools and drugs: recent developments. ChemBioChem. 2003; 4:963–971. 14523912 10.1002/cbic.200300648

[24] Brody E.N., Gold L. Aptamers as therapeutic and diagnostic agents. Rev. Mol. Biotechnol. 2000; 74:5–13. 10.1016/s1389-0352(99)00004-5 10943568

[25] Kolpashchikov D.M. Binary probes for nucleic acid analysis. Chem. Rev. 2010; 110:4709–4723. 20583806 10.1021/cr900323b

[26] Wilson C., Keefe A. Building oligonucleotide therapeutics using non-natural chemistries. Curr. Opin. Chem. Biol. 2006; 10:607–614. 17049298 10.1016/j.cbpa.2006.10.001

[27] Röthlisberger P., Hollenstein M. Aptamer chemistry. Adv. Drug Deliv. Rev. 2018; 134:3–21. 29626546 10.1016/j.addr.2018.04.007

[28] Dunn M.R., Jimenez R.M., Chaput J.C. Analysis of aptamer discovery and technology. Nat. Rev. Chem. 2017; 1:0076.

[29] Pfeiffer F., Mayer G. Selection and biosensor application of aptamers for small molecules. Front. Chem. 2016; 4:1–21. 27379229 10.3389/fchem.2016.00025 PMC4908669

[30] Munzar J.D., Ng A., Juncker D. Duplexed aptamers: history, design, theory, and application to biosensing. Chem. Soc. Rev. 2019; 48:1390–1419. 30707214 10.1039/c8cs00880a

[31] Lakhin A.V., Tarantul V.Z., Gening L.V. Aptamers: problems, solutions and prospects. Acta Naturae. 2013; 5:34–43. 24455181 PMC3890987

[32] Shekhawat S.S., Ghosh I. Split-protein systems: beyond binary protein–protein interactions. Curr. Opin. Chem. Biol. 2011; 15:789–797. 22070901 10.1016/j.cbpa.2011.10.014 PMC3237955

[33] Huizenga D.E., Szostak J.W. A DNA aptamer that binds adenosine and ATP. Biochemistry. 1995; 34:656–665. 7819261 10.1021/bi00002a033

[34] Tang J., Shi H., He X., Lei Y., Guo Q., Wang K., Yan L., He D. Tumor cell-specific split aptamers: target-driven and temperature-controlled self-assembly on the living cell surface. Chem. Commun. 2016; 52:1482–1485. 10.1039/c5cc08977h 26660498

[35] Chen A., Yan M., Yang S. Split aptamers and their applications in sandwich aptasensors. Trends Anal. Chem. 2016; 80:581–593.

[36] Davie E.W., Kulman J.D. An overview of the structure and the function of thrombin. Semin. Thromb. Hemost. 2006; 32:3–15. 10.1055/s-2006-939550 16673262

[37] Fenton J.W., Aronson D.L., Young A.M., Finlayson J.S. Human thrombins: production, evaluation and properties of alpha-thrombin. J. Biol. Chem. 1977; 252:3587–3598. 16908

[38] Mohammed S.F., Whitworth C., Chuang H.Y., Lundblad R.L., Mason R.G. Multiple active forms of thrombin: binding to platelets and effects on platelet function. Proc. Natl. Acad. Sci. U.S.A. 1976; 73:1660–1663. 1064039 10.1073/pnas.73.5.1660 PMC430359

[39] Macaya R.F., Schultze P., Smith F.W., Roe J.A., Feigon J. Thrombin-binding DNA aptamer forms a unimolecular quadruplex structure in solution. Proc. Natl. Acad. Sci. U.S.A. 1993; 90:3745–3749. 8475124 10.1073/pnas.90.8.3745 PMC46378

[40] Tsiang M., Gibbs C.S., Griffin L.C., Dunn K.E., Leung L.K. Selection of a suppressor mutation that restores affinity of an oligonucleotide inhibitor for thrombin using in vitro genetics. J. Biol. Chem. 1995; 270:19370–19376. 7642616 10.1074/jbc.270.33.19370

[41] Wang K.Y., Krawczyk S.H., Bischofberger N., Swaminathan S., Bolton P.H. The tertiary structure of a DNA aptamer which binds to and inhibits thrombin determines activity. Biochemistry. 1993; 32:11285–11292. 8218193 10.1021/bi00093a004

[42] Russo Krauss I., Pica A., Merlino A., Mazzarella L., Sica F. Duplex–quadruplex motifs in a peculiar structural organization cooperatively contribute to thrombin binding of a DNA aptamer. Acta Crystallogr. 2013; 69:2403–2411. 10.1107/S0907444913022269 24311581

[43] Macaya R.F., Waldron J.A., Beutel B.A., Gao H., Joesten M.E., Yang M., Patel R., Bertelsen A.H., Cook A.F. Structural and functional characterization of potent antithrombotic oligonucleotides possessing both quadruplex and duplex motifs. Biochemistry. 1995; 34:4478–4492. 7703261 10.1021/bi00013a041

[44] Tasset D.M., Kubik M.F., Steiner W. Oligonucleotide inhibitors of human thrombin that bind distinct epitopes. J. Mol. Biol. 1997; 272:688–698. 9368651 10.1006/jmbi.1997.1275

[45] Stubbs M.T., Bode W. The clot thickens: clues provided by thrombin structure. Trends Biochem. Sci. 1995; 20:23–28. 7878739 10.1016/s0968-0004(00)88945-8

[46] Kelly J.A., Feigon J., Yeates T.O. Reconciliation of the X-ray and NMR structures of thrombin-binding aptamer d(GGTTGGTGTGGTTGG). J. Mol. Biol. 1996; 256:417–422. 8604127 10.1006/jmbi.1996.0097

[47] Hamaguchi N., Ellington A., Stanton M. Aptamer beacons for the direct detection of proteins. Anal. Biochem. 2001; 294:126–131. 11444807 10.1006/abio.2001.5169

[48] Li J.J., Fang X., Tan W. Molecular aptamer beacons for real-time protein recognition. Biochem. Biophys. Res. Commun. 2002; 292:31–40. 11890667 10.1006/bbrc.2002.6581

[49] Deng B., Lin Y., Wang C., Li F., Wang Z., Zhang H., Li X.-F., Le X.C. Aptamer binding assays for proteins: thrombin example—a review. Anal. Chim. Acta. 2014; 837:1–15. 25000852 10.1016/j.aca.2014.04.055

[50] Liu X., Shi L., Hua X., Huang Y., Su S., Fan Q., Wang L., Huang W. Target-Induced conjunction of split aptamer fragments and assembly with a Water-Soluble conjugated polymer for improved protein detection. ACS Appl. Mater. Interfaces. 2014; 6:3406–3412. 24512085 10.1021/am405550j

[51] Liu X., Yang Y., Hua X., Feng X., Su S., Huang Y., Fan Q., Wang L., Huang W. An improved turn-on aptasensor for Thrombin detection using split aptamer fragments and graphene oxide. Chin. J. Chem. 2015; 33:981–986.

[52] Liu Y., Dong X., Chen P. Biological and chemical sensors based on graphene materials. Chem. Soc. Rev. 2012; 41:2283–2307. 22143223 10.1039/c1cs15270j

[53] Chen D., Feng H., Li J. Graphene oxide: preparation, functionalization, and electrochemical applications. Chem. Rev. 2012; 112:6027–6053. 22889102 10.1021/cr300115g

[54] Duan W., Wang X., Wang H., Li F. Fluorescent and colorimetric dual-mode aptasensor for thrombin detection based on target-induced conjunction of split aptamer fragments. Talanta. 2018; 180:76–80. 29332836 10.1016/j.talanta.2017.12.033

[55] Wang H., Wang Y., Jin J., Yang R. Gold Nanoparticle-Based colorimetric and “Turn-On” fluorescent probe for Mercury(II) ions in aqueous solution. Anal. Chem. 2008; 80:9021–9028. 19551976 10.1021/ac801382k

[56] Chen J., Zhang J., Li J., Yang H.-H., Fu F., Chen G. An ultrasensitive signal-on electrochemical aptasensor via target-induced conjunction of split aptamer fragments. Biosens. Bioelectron. 2010; 25:996–1000. 19818593 10.1016/j.bios.2009.09.015

[57] Liu Z., Zhang W., Hu L., Li H., Zhu S., Xu G. Label-Free and Signal-On electrochemiluminescence aptasensor for ATP based on Target-Induced linkage of split aptamer fragments by using [Ru(phen)3]2+ intercalated into Double-Strand DNA as a probe. Chem. – Eur. J. 2010; 16:13356–13359. 21053209 10.1002/chem.201001736

[58] Lin Z., Chen L., Zhu X., Qiu B., Chen G. Signal-on electrochemiluminescence biosensor for thrombin based on target-induced conjunction of split aptamer fragments. Chem. Commun. 2010; 46:5563–5565. 10.1039/c0cc00932f 20532276

[59] Fan T., Du Y., Yao Y., Wu J., Meng S., Luo J., Zhang X., Yang D., Wang C., Qian Y. et al. Rolling circle amplification triggered poly adenine-gold nanoparticles production for label-free electrochemical detection of thrombin. Sens. Actuat. B Chem. 2018; 266:9–18.

[60] Pei H., Li F., Wan Y., Wei M., Liu H., Su Y., Chen N., Huang Q., Fan C. Designed diblock oligonucleotide for the synthesis of spatially isolated and highly hybridizable functionalization of DNA–gold nanoparticle nanoconjugates. J. Am. Chem. Soc. 2012; 134:11876–11879. 22799460 10.1021/ja304118z

[61] Zhao B., Shen J., Chen S., Wang D., Li F., Mathur S., Song S., Fan C. Gold nanostructures encoded by non-fluorescent small molecules in polyA-mediated nanogaps as universal SERS nanotags for recognizing various bioactive molecules. Chem. Sci. 2014; 5:4460–4466.

[62] Lohmann K. Über die Pyrophosphatfraktion im Muskel. Naturwissenschaften. 1929; 17:624–625.

[63] Fiske C.H., Subbarow Y. Phosphorus compounds of muscle and liver. Science. 1929; 70:381–382. 17756191 10.1126/science.70.1816.381.b

[64] Sassanfar M., Szostak J.W. An RNA motif that binds ATP. Nature. 1993; 364:550–553. 7687750 10.1038/364550a0

[65] Liao D., Jiao H., Wang B., Lin Q., Yu C. KF polymerase-based fluorescence aptasensor for the label-free adenosine detection. Analyst. 2012; 137:978–982. 22183639 10.1039/c2an15809d

[66] He X., Li Z., Jia X., Wang K., Yin J. A highly selective sandwich-type FRET assay for ATP detection based on silica coated photon upconverting nanoparticles and split aptamer. Talanta. 2013; 111:105–110. 23622532 10.1016/j.talanta.2013.02.050

[67] Wang M., Chen J., Su D., Wang G., Su X. Split aptamer based sensing platform for adenosine deaminase detection by fluorescence resonance energy transfer. Talanta. 2019; 198:1–7. 30876536 10.1016/j.talanta.2019.01.041

[68] Tang X., Wu K., Zhao H., Chen M., Ma C. A label-free fluorescent assay for the rapid and sensitive detection of adenosine deaminase activity and inhibition. Sensors. 2018; 18:2441. 10.3390/s18082441 PMC6111851 30060448

[69] Jin F., Lian Y., Li J., Zheng J., Hu Y., Liu J., Huang J., Yang R. Molecule-binding dependent assembly of split aptamer and γ-cyclodextrin: a sensitive excimer signaling approach for aptamer biosensors. Anal. Chim. Acta. 2013; 799:44–50. 24091373 10.1016/j.aca.2013.08.012

[70] Zhang H., Liu Y., Zhang K., Ji J., Liu J., Liu B. Single molecule fluorescent colocalization of split aptamers for ultrasensitive detection of biomolecules. Anal. Chem. 2018; 90:9315–9321. 30003776 10.1021/acs.analchem.8b01916

[71] Wang J., Wang Y., Liu S., Wang H., Zhang X., Song X., Huang J. Duplex featured polymerase-driven concurrent strategy for detecting of ATP based on endonuclease-fueled feedback amplification. Anal. Chim. Acta. 2019; 1060:79–87. 30902334 10.1016/j.aca.2019.01.047

[72] You J., You Z., Xu X., Ji J., Lu T., Xia Y., Wang L., Zhang L., Du S. A split aptamer-labeled ratiometric fluorescent biosensor for specific detection of adenosine in human urine. Microchim. Acta. 2019; 186:43–50. 10.1007/s00604-018-3162-2 30569231

[73] Ma Y., Geng F., Wang Y., Xu M., Shao C., Qu P., Zhang Y., Ye B. Novel strategy to improve the sensing performances of split ATP aptamer based fluorescent indicator displacement assay through enhanced molecular recognition. Biosens. Bioelectron. 2019; 134:36–41. 30954924 10.1016/j.bios.2019.03.047

[74] Zhou C., Yu Z., Yu W., Liu H., Zhang H., Guo C. Split aptamer-based detection of adenosine triphosphate using surface enhanced Raman spectroscopy and two kinds of gold nanoparticles. Microchim. Acta. 2019; 186:251. 10.1007/s00604-019-3356-2 30895481

[75] Zeng S., Baillargeat D., Ho H.-P., Yong K.-T. Nanomaterials enhanced surface plasmon resonance for biological and chemical sensing applications. Chem. Soc. Rev. 2014; 43:3426–3452. 24549396 10.1039/c3cs60479a

[76] Homola J., Yee S.S., Gauglitz G. Surface plasmon resonance sensors: review. Sens. Actuat. B Chem. 1999; 54:3–15.

[77] Li F., Zhang J., Cao X., Wang L., Li D., Song S., Ye B., Fan C. Adenosine detection by using gold nanoparticles and designed aptamer sequences. Analyst. 2009; 134:1355–1360. 19562201 10.1039/b900900k

[78] Melaine F., Coilhac C., Roupioz Y., Buhot A. A nanoparticle-based thermo-dynamic aptasensor for small molecule detection. Nanoscale. 2016; 8:16947–16954. 27714066 10.1039/c6nr04868d

[79] Sergelen K., Liedberg B., Knoll W., Dostálek J. A surface plasmon field-enhanced fluorescence reversible split aptamer biosensor. Analyst. 2017; 142:2995–3001. 28744534 10.1039/c7an00970d

[80] Lu C., Saint-Pierre C., Gasparutto D., Roupioz Y., Peyrin E., Buhot A. Linear chain formation of split-aptamer dimers on surfaces triggered by adenosine. Langmuir. 2017; 33:12785–12792. 29035542 10.1021/acs.langmuir.7b02104

[81] Park J.-H., Byun J.-Y., Shim W.-B., Kim S.U., Kim M.-G. High-sensitivity detection of ATP using a localized surface plasmon resonance (LSPR) sensor and split aptamers. Biosens. Bioelectron. 2015; 73:26–31. 26042875 10.1016/j.bios.2015.05.043

[82] Wang Q., Huang J., Yang X., Wang K., He L., Li X., Xue C. Surface plasmon resonance detection of small molecule using split aptamer fragments. Sens. Actuat. B Chem. 2011; 156:893–898.

[83] Stojanovic M.N., de Prada P., Landry D.W. Fluorescent sensors based on aptamer self-assembly. J. Am. Chem. Soc. 2000; 122:11547–11548. 29048887 10.1021/ja0022223

[84] Alkhamis O., Canoura J., Yu H., Liu Y., Xiao Y. Innovative engineering and sensing strategies for aptamer-based small-molecule detection. Trends Anal. Chem. 2019; 121:115699. 10.1016/j.trac.2019.115699 PMC7450712 32863483

[85] Cekan P., Jonsson E.O., Sigurdsson S.Th Folding of the cocaine aptamer studied by EPR and fluorescence spectroscopies using the bifunctional spectroscopic probe C. Nucleic Acids Res. 2009; 37:3990–3995. 19406921 10.1093/nar/gkp277 PMC2709570

[86] Stojanovic M.N., De Prada P., Landry D.W. Aptamer-based folding fluorescent sensor for cocaine. J. Am. Chem. Soc. 2001; 123:4928–4931. 11457319 10.1021/ja0038171

[87] Wu C., Yan L., Wang C., Lin H., Wang C., Chen X., Yang C.J. A general excimer signaling approach for aptamer sensors. Biosens. Bioelectron. 2010; 25:2232–2237. 20378328 10.1016/j.bios.2010.02.030

[88] He J.-L., Wu Z.-S., Zhou H., Wang H.-Q., Jiang J.-H., Shen G.-L., Yu R.-Q. Fluorescence aptameric sensor for strand displacement amplification detection of Cocaine. Anal. Chem. 2010; 82:1358–1364. 20078091 10.1021/ac902416u

[89] Tang Y., Long F., Gu C., Wang C., Han S., He M. Reusable split-aptamer-based biosensor for rapid detection of cocaine in serum by using an all-fiber evanescent wave optical biosensing platform. Anal. Chim. Acta. 2016; 933:182–188. 27497011 10.1016/j.aca.2016.05.021

[90] Zhang Y., Sun Z., Tang L., Zhang H., Zhang G.-J. Aptamer based fluorescent cocaine assay based on the use of graphene oxide and exonuclease III-assisted signal amplification. Microchim. Acta. 2016; 183:2791–2797.

[91] Neves M.A.D., Reinstein O., Johnson P.E. Defining a stem length-dependent binding mechanism for the cocaine-binding aptamer. A combined NMR and calorimetry study. Biochemistry. 2010; 49:8478–8487. 20735071 10.1021/bi100952k

[92] Morris F.D., Peterson E.M., Heemstra J.M., Harris J.M. Single-molecule kinetic investigation of cocaine-dependent split-aptamer assembly. Anal. Chem. 2018; 90:12964–12970. 30280568 10.1021/acs.analchem.8b03637

[93] Li Q., Wang Y.-D., Shen G.-L., Tang H., Yu R.-Q., Jiang J.-H. Split aptamer mediated endonuclease amplification for small-molecule detection. Chem. Commun. 2015; 51:4196–4199. 10.1039/c5cc00390c 25672262

[94] Zuo X., Xiao Y., Plaxco K.W. High specificity, electrochemical sandwich assays based on single aptamer sequences and suitable for the direct detection of small-molecule targets in blood and other complex matrices. J. Am. Chem. Soc. 2009; 131:6944–6945. 19419171 10.1021/ja901315w PMC2994717

[95] Zhang J., Wang L., Pan D., Song S., Boey F.Y.C., Zhang H., Fan C. Visual cocaine detection with gold nanoparticles and rationally engineered aptamer structures. Small. 2008; 4:1196–1200. 18651718 10.1002/smll.200800057

[96] Abnous K., Danesh N.M., Ramezani M., Taghdisi S.M., Emrani A.S. A novel amplified double-quenching aptasensor for cocaine detection based on split aptamer and silica nanoparticles. Anal. Methods. 2018; 10:3232–3236.

[97] Kolb H.C., Finn M.G., Sharpless K.B. Click chemistry: diverse chemical function from a few good reactions. Angew. Chem. Int. Ed. 2001; 40:2004–2021. 10.1002/1521-3773(20010601)40:11<2004::AID-ANIE2004>3.0.CO;2-5 11433435

[98] El-Sagheer A.H., Brown T. Click chemistry with DNA. Chem. Soc. Rev. 2010; 39:1388–1405. 20309492 10.1039/b901971p

[99] Gorska K., Winssinger N. Reactions templated by nucleic acids: more ways to translate oligonucleotide‐based instructions into emerging function. Angew. Chem. Int. Ed. 2013; 52:6820–6843. 10.1002/anie.201208460 23794204

[100] Sharma A.K., Heemstra J.M. Small-molecule-dependent split aptamer ligation. J. Am. Chem. Soc. 2011; 133:12426–12429. 21761903 10.1021/ja205518e

[101] Sachan A., Ilgu M., Kempema A., Kraus G.A., Nilsen-Hamilton M. Specificity and ligand affinities of the cocaine aptamer: impact of structural features and physiological NaCl. Anal. Chem. 2016; 88:7715–7723. 27348073 10.1021/acs.analchem.6b01633

[102] Spiropulos N.G., Heemstra J.M. Templating effect in DNA proximity ligation enables use of non-bioorthogonal chemistry in biological fluids. Artif. DNA PNA XNA. 2012; 3:123–128. 23370267 10.4161/adna.23842 PMC3581511

[103] Sharma A.K., Kent A.D., Heemstra J.M. Enzyme-linked small-molecule detection using split aptamer ligation. Anal. Chem. 2012; 84:6104–6109. 22715870 10.1021/ac300997q

[104] Yu H., Canoura J., Guntupalli B., Lou X., Xiao Y. A cooperative-binding split aptamer assay for rapid, specific and ultra-sensitive fluorescence detection of cocaine in saliva. Chem. Sci. 2017; 8:131–141. 28451157 10.1039/c6sc01833e PMC5308383

[105] Roncancio D., Yu H., Xu X., Wu S., Liu R., Debord J., Lou X., Xiao Y. A label-free aptamer-fluorophore assembly for rapid and specific detection of cocaine in Biofluids. Anal. Chem. 2014; 86:11100–11106. 25342426 10.1021/ac503360n

[106] Yu H., Canoura J., Guntupalli B., Alkhamis O., Xiao Y. Sensitive detection of small-molecule targets using cooperative binding split aptamers and enzyme-assisted target recycling. Anal. Chem. 2018; 90:1748–1758. 29294287 10.1021/acs.analchem.7b03625 PMC5803384

[107] Reinstein O., Yoo M., Han C., Palmo T., Beckham S.A., Wilce M.C.J., Johnson P.E. Quinine binding by the cocaine-binding aptamer. Thermodynamic and hydrodynamic analysis of high-affinity binding of an off-target ligand. Biochemistry. 2013; 52:8652–8662. 24175947 10.1021/bi4010039

[108] Pei R., Shen A., Olah M.J., Stefanovic D., Worgall T., Stojanovic M.N. High-resolution cross-reactive array for alkaloids. Chem. Commun. 2009; 22:3193–3195. 10.1039/b900001a 19587910

[109] Neves M.A.D., Slavkovic S., Reinstein O., Shoara A.A., Johnson P.E. A proof of concept application of aptachain: ligand-induced self-assembly of a DNA aptamer. RSC Adv. 2019; 9:1690–1695. 10.1039/c8ra07462c PMC9059725 35518030

[110] Wang J., Cheng W., Meng F., Yang M., Pan Y., Miao P. Hand-in-hand RNA nanowire-based aptasensor for the detection of theophylline. Biosens. Bioelectron. 2018; 101:153–158. 29065340 10.1016/j.bios.2017.10.025

[111] Silverman S.K. Deoxyribozymes: DNA catalysts for bioorganic chemistry. Org. Biomol. Chem. 2004; 2:2701–2706. 15455136 10.1039/B411910J

[112] Silverman S.K. In vitro selection, characterization, and application of deoxyribozymes that cleave RNA. Nucleic Acids Res. 2005; 33:6151–6163. 16286368 10.1093/nar/gki930 PMC1283523

[113] Lan T., Furuya K., Lu Y. A highly selective lead sensor based on a classic lead DNAzyme. Chem. Commun. 2010; 46:3896–3898. 10.1039/b926910j PMC3071848 20407665

[114] Santoro S.W., Joyce G.F. A general purpose RNA-cleaving DNA enzyme. Proc. Natl. Acad. Sci. 1997; 94:4262–4266. 9113977 10.1073/pnas.94.9.4262 PMC20710

[115] Cuenoud B., Szostak J.W. A DNA metalloenzyme with DNA ligase activity. Nature. 1995; 375:611–614. 7791880 10.1038/375611a0

[116] Silverman S.K. Catalytic DNA: scope, applications, and biochemistry of deoxyribozymes. Trends Biochem. Sci. 2016; 41:595–609. 27236301 10.1016/j.tibs.2016.04.010 PMC4930396

[117] Baum D.A., Silverman S.K. Deoxyribozymes: useful DNA catalysts in vitro and in vivo. Cell. Mol. Life Sci. 2008; 65:2156–2174. 18373062 10.1007/s00018-008-8029-y PMC7079777

[118] Gong L., Zhao Z., Lv Y.-F., Huan S.-Y., Fu T., Zhang X.-B., Shen G.-L., Yu R.-Q. DNAzyme-based biosensors and nanodevices. Chem. Commun. 2015; 51:979–995. 10.1039/c4cc06855f 25336076

[119] Höbartner C., Silverman S.K. Recent advances in DNA catalysis. Biopolymers. 2007; 87:279–292. 17647280 10.1002/bip.20813

[120] Willner I., Shlyahovsky B., Zayats M., Willner B. DNAzymes for sensing, nanobiotechnology and logic gate applications. Chem. Soc. Rev. 2008; 37:1153–1165. 18497928 10.1039/b718428j

[121] Mokany E., Bone S.M., Young P.E., Doan T.B., Todd A.V. MNAzymes, a versatile new class of nucleic acid enzymes that can function as biosensors and molecular switches. J. Am. Chem. Soc. 2010; 132:1051–1059. 20038095 10.1021/ja9076777 PMC2808728

[122] Mokany E., Tan Y.L., Bone S.M., Fuery C.J., Todd A.V. MNAzyme qPCR with superior multiplexing capacity. Clin. Chem. 2013; 59:419–426. 23232065 10.1373/clinchem.2012.192930

[123] Ruble B.K., Richards J.L., Cheung-Lau J.C., Dmochowski I.J. Mismatch discrimination and efficient photomodulation with split 10–23 DNAzymes. Inorganica Chim. Acta. 2012; 380:386–391. 22544974 10.1016/j.ica.2011.10.068 PMC3337724

[124] Huang P.-J.J., Liu J. Two Pb^2+^-specific DNAzymes with opposite trends in split-site-dependent activity. Chem. Commun. 2014; 50:4442–4444. 10.1039/c4cc00864b 24643441

[125] Smith A.L., Kolpashchikov D.M. Divide and control: comparison of split and switch hybridization sensors. ChemistrySelect. 2017; 2:5427–5431. 29372178 10.1002/slct.201701179 PMC5777618

[126] Xie Y., Niu F., Yu A., Lai G. Proximity binding-triggered assembly of two MNAzymes for catalyzed release of G-Quadruplex DNAzymes and an ultrasensitive homogeneous bioassay of platelet-derived growth factor. Anal. Chem. 2020; 92:593–598. 31855409 10.1021/acs.analchem.9b05002

[127] Lu L.-M., Zhang X.-B., Kong R.-M., Yang B., Tan W. A ligation-triggered DNAzyme cascade for amplified fluorescence detection of biological small molecules with zero-background signal. J. Am. Chem. Soc. 2011; 133:11686–11691. 21662240 10.1021/ja203693b PMC5512710

[128] Sheng A., Su L., Jalalah M., Al-Assiri M.S., Harraz F.A., Zhang J. Hydrazone chemistry assisted DNAzyme for the analysis of double targets. Chem. Commun. 2020; 56:695–698. 10.1039/c9cc09389c 31848532

[129] Chen J., Pan J., Chen S. A naked-eye colorimetric sensor for Hg^2+^ monitoring with cascade signal amplification based on target-induced conjunction of split DNAzyme fragments. Chem. Commun. 2017; 53:10224–10227. 10.1039/c7cc05445a 28861569

[130] Kim H.N., Ren W.X., Kim J.S., Yoon J. Fluorescent and colorimetric sensors for detection of lead, cadmium, and mercury ions. Chem. Soc. Rev. 2012; 41:3210–3244. 22184584 10.1039/c1cs15245a

[131] Wu Y., Huang J., Yang X., Yang Y., Quan K., Xie N., Li J., Ma C., Wang K. Gold nanoparticle loaded split-DNAzyme probe for amplified miRNA detection in living cells. Anal. Chem. 2017; 89:8377–8383. 28718626 10.1021/acs.analchem.7b01632

[132] Travascio P., Li Y., Sen D. DNA-enhanced peroxidase activity of a DNA aptamer-hemin complex. Chem. Biol. 1998; 5:505–517. 9751647 10.1016/s1074-5521(98)90006-0

[133] Deng M., Zhang D., Zhou Y., Zhou X. Highly effective colorimetric and visual detection of nucleic acids using an asymmetrically split Peroxidase DNAzyme. J. Am. Chem. Soc. 2008; 130:13095–13102. 18763776 10.1021/ja803507d

[134] Nakayama S., Sintim H.O. Colorimetric split G-Quadruplex probes for nucleic acid sensing: improving reconstituted DNAzyme's catalytic efficiency via probe remodeling. J. Am. Chem. Soc. 2009; 131:10320–10333. 19621970 10.1021/ja902951b

[135] Deng M., Feng S., Luo F., Wang S., Sun X., Zhou X., Zhang X.-L. Visual detection of rpoB mutations in Rifampin-resistant mycobacterium tuberculosis strains by use of an asymmetrically split peroxidase DNAzyme. J. Clin. Microbiol. 2012; 50:3443–3450. 22875898 10.1128/JCM.01292-12 PMC3486259

[136] Shahbazi N., Hosseinkhani S., Ranjbar B. A facile and rapid aptasensor based on split peroxidase DNAzyme for visual detection of carcinoembryonic antigen in saliva. Sens. Actuat. B Chem. 2017; 253:794–803.

[137] Zhu D., Luo J., Rao X., Zhang J., Cheng G., He P., Fang Y. A novel optical thrombin aptasensor based on magnetic nanoparticles and split DNAzyme. Anal. Chim. Acta. 2012; 711:91–96. 22152801 10.1016/j.aca.2011.10.053

[138] Zhang X.F., Li N., Ling Y., Tang L., Li N.B., Luo H.Q. Linked bridge hybridizing-induced split G-quadruplex DNA machine and its application to uracil-DNA glycosylase detection. Sens. Actuat. B Chem. 2018; 255:2589–2594.

[139] Zhou D., Wu W., Li Q., Pan J., Chen J. A label-free and enzyme-free aptasensor for visual Cd^2+^ detection based on split DNAzyme fragments. Anal. Methods. 2019; 11:3546–3551.

[140] Zhou W., Ding J., Liu J. Splitting a DNAzyme enables a Na^+^-dependent FRET signal from the embedded aptamer. Org. Biomol. Chem. 2017; 15:6959–6966. 28792040 10.1039/c7ob01709j

[141] Zhou X.-H., Kong D.-M., Shen H.-X. Ag^+^ and cysteine quantitation based on G-Quadruplex−Hemin DNAzymes disruption by Ag^+^. Anal. Chem. 2010; 82:789–793. 20039758 10.1021/ac902421u

[142] Xu J., Lee E.-S., Gye M.C., Kim Y.-P. Rapid and sensitive determination of bisphenol A using aptamer and split DNAzyme. Chemosphere. 2019; 228:110–116. 31026631 10.1016/j.chemosphere.2019.04.110

[143] Luo Y., Yu H., Alkhamis O., Liu Y., Lou X., Yu B., Xiao Y. Label-free, visual detection of small molecules using highly target-responsive multimodule split aptamer constructs. Anal. Chem. 2019; 91:7199–7207. 31050407 10.1021/acs.analchem.9b00507 PMC6615563

[144] Hou T., Li C., Wang X., Zhao C., Li F. Label-free colorimetric detection of coralyne utilizing peroxidase-like split G-quadruplex DNAzyme. Anal. Methods. 2013; 5:4671–4674.

[145] Cui X., Li R., Liu X., Wang J., Leng X., Song X., Pei Q., Wang Y., Liu S., Huang J. Low-background and visual detection of antibiotic based on target-activated colorimetric split peroxidase DNAzyme coupled with dual nicking enzyme signal amplification. Anal. Chim. Acta. 2018; 997:1–8. 29149989 10.1016/j.aca.2017.10.009

[146] Zhang R., Wang Y., Qu X., Li S., Zhao Y., Zhang F., Liu S., Huang J., Yu J. A label-free electrochemical platform for antibiotics detection based on cascade enzymatic amplification coupled with split G-quadruplex DNAzyme. Analyst. 2019; 144:4995–5002. 31328736 10.1039/c9an00857h

[147] Xiao X., Zhu L., He W., Luo Y., Xu W. Functional nucleic acids tailoring and its application. Trends Anal. Chem. 2019; 118:138–157.

[148] Sun Y., Yuan B., Deng M., Wang Q., Huang J., Guo Q., Liu J., Yang X., Wang K. A light-up fluorescence assay for tumor cell detection based on bifunctional split aptamers. Analyst. 2018; 143:3579–3585. 29999048 10.1039/c8an01008k

[149] Yuan B., Zhou Y., Guo Q., Wang K., Yang X., Meng X., Wan J., Tan Y., Huang Z., Xie Q. et al. A signal-on split aptasensor for highly sensitive and specific detection of tumor cells based on FRET. Chem. Commun. 2016; 52:1590–1593. 10.1039/c5cc08060f 26661391

[150] Lei Y., Tang J., Shi H., Ye X., He X., Xu F., Yan L., Qiao Z., Wang K. Nature-Inspired smart DNA Nanodoctor for activatable in vivo cancer imaging and in situ drug release based on recognition-triggered assembly of split aptamer. Anal. Chem. 2016; 88:11699–11706. 27807977 10.1021/acs.analchem.6b03283

[151] Kikuchi N., Reed A., Gerasimova Y.V., Kolpashchikov D.M. Split Dapoxyl aptamer for sequence-selective analysis of nucleic acid sequence based amplification amplicons. Anal. Chem. 2019; 91:2667–2671. 30680988 10.1021/acs.analchem.8b03964 PMC7720896

[152] Fatin M.F., Rahim Ruslinda A., Gopinath S.C.B., Arshad M.K.Md High-performance interactive analysis of split aptamer and HIV-1 Tat on multiwall carbon nanotube-modified field-effect transistor. Int. J. Biol. Macromol. 2019; 125:414–422. 30529550 10.1016/j.ijbiomac.2018.12.066

[153] Belal A.S.F., Ismail A., Elnaggar M.M., Belal T.S. Click chemistry inspired copper sulphide nanoparticle-based fluorescence assay of kanamycin using DNA aptamer. Spectrochim. Acta A. 2018; 205:48–54. 10.1016/j.saa.2018.07.011 30007899

[154] Li X.-H., Sun W.-M., Wu J., Gao Y., Chen J.-H., Chen M., Ou Q.-S. An ultrasensitive fluorescence aptasensor for carcino-embryonic antigen detection based on fluorescence resonance energy transfer from upconversion phosphors to Au nanoparticles. Anal. Methods. 2018; 10:1552–1559.

[155] Zhang K., Lv S., Lu M., Tang D. Photoelectrochemical biosensing of disease marker on p-type Cu-doped Zn0.3Cd0.7S based on RCA and exonuclease III amplification. Biosens. Bioelectron. 2018; 117:590–596. 30005378 10.1016/j.bios.2018.07.001

[156] Chen X., Lan J., Liu Y., Li L., Yan L., Xia Y., Wu F., Li C., Li S., Chen J. A paper-supported aptasensor based on upconversion luminescence resonance energy transfer for the accessible determination of exosomes. Biosens. Bioelectron. 2018; 102:582–588. 29241062 10.1016/j.bios.2017.12.012

[157] Liu X., Li X., Lu Y., Cao J., Li F. A split aptamer-based imaging solution for the visualization of latent fingerprints. Anal. Methods. 2018; 10:2281–2286.

[158] Alila K.O., Baum D.A. Modulation of an RNA-branching deoxyribozyme by a small molecule. Chem. Commun. 2011; 47:3227–3229. 10.1039/c0cc04971a 21258742

[159] Huang J., He Y., Yang X., Wang K., Quan K., Lin X. Split aptazyme-based catalytic molecular beacons for amplified detection of adenosine. Analyst. 2014; 139:2994–2997. 24807151 10.1039/c4an00454j

[160] Liu J., Lu Y. Adenosine-dependent assembly of aptazyme-functionalized gold nanoparticles and its application as a colorimetric biosensor. Anal. Chem. 2004; 76:1627–1632. 15018560 10.1021/ac0351769

[161] Ausländer S., Fuchs D., Hürlemann S., Ausländer D., Fussenegger M. Engineering a ribozyme cleavage-induced split fluorescent aptamer complementation assay. Nucleic Acids Res. 2016; 44:e94. 26939886 10.1093/nar/gkw117 PMC4889925

[162] Li J., He G., Wang B., Shi L., Gao T., Li G. Fabrication of reusable electrochemical biosensor and its application for the assay of α-glucosidase activity. Anal. Chim. Acta. 2018; 1026:140–146. 29852990 10.1016/j.aca.2018.04.015

[163] Liu H., Yang J., Li Z., Xiao L., Aryee A.A., Sun Y., Yang R., Meng H., Qu L., Lin Y. et al. Hydrogen-bond-induced emission of carbon dots for wash-free nucleus imaging. Anal. Chem. 2019; 91:9259–9265. 31204808 10.1021/acs.analchem.9b02147

[164] Shimomura O., Johnson F.H., Saiga Y. Extraction, purification and properties of aequorin, a bioluminescent protein from the luminous hydromedusan, aequorea. J. Cell. Comp. Physiol. 1962; 59:223–239. 13911999 10.1002/jcp.1030590302

[165] Meech S.R. Excited state reactions in fluorescent proteins. Chem. Soc. Rev. 2009; 38:2922–2934. 19771336 10.1039/b820168b

[166] Grate D., Wilson C. Laser-mediated, site-specific inactivation of RNA transcripts. Proc. Natl. Acad. Sci. U.S.A. 1999; 96:6131–6136. 10339553 10.1073/pnas.96.11.6131 PMC26847

[167] Kraus G., Jeon I., Nilsen-Hamilton M., Awad A., Banerjee J., Parvin B. Fluorinated analogs of malachite green: synthesis and toxicity. Molecules. 2008; 13:986–994. 18463600 10.3390/molecules13040986 PMC6245195

[168] Constantin T.P., Silva G.L., Robertson K.L., Hamilton T.P., Fague K., Waggoner A.S., Armitage B.A. Synthesis of new fluorogenic cyanine dyes and incorporation into RNA fluoromodules. Org. Lett. 2008; 10:1561–1564. 18338898 10.1021/ol702920e

[169] Sando S., Narita A., Aoyama Y. Light-up Hoechst–DNA aptamer pair: generation of an aptamer-selective fluorophore from a conventional DNA-Staining dye. ChemBioChem. 2007; 8:1795–1803. 17806095 10.1002/cbic.200700325

[170] Sando S., Narita A., Hayami M., Aoyama Y. Transcription monitoring using fused RNA with a dye-binding light-up aptamer as a tag: a blue fluorescent RNA. Chem. Commun. 2008; 3858–3860.doi:10.1039/b808449a. 10.1039/b808449a 18726014

[171] Dolgosheina E.V., Jeng S.C.Y., Panchapakesan S.S.S., Cojocaru R., Chen P.S.K., Wilson P.D., Hawkins N., Wiggins P.A., Unrau P.J. RNA mango aptamer-fluorophore: a bright, high-affinity complex for RNA labeling and tracking. ACS Chem. Biol. 2014; 9:2412–2420. 25101481 10.1021/cb500499x

[172] Sheng L., Lu Y., Deng S., Liao X., Zhang K., Ding T., Gao H., Liu D., Deng R., Li J. A transcription aptasensor: amplified, label-free and culture-independent detection of foodborne pathogens *via* light-up RNA aptamers. Chem. Commun. 2019; 55:10096–10099. 10.1039/c9cc05036a 31380872

[173] Stojanovic M.N., Kolpashchikov D.M. Modular aptameric sensors. J. Am. Chem. Soc. 2004; 126:9266–9270. 15281816 10.1021/ja032013t

[174] Kolpashchikov D.M. Binary malachite green aptamer for fluorescent detection of nucleic acids. J. Am. Chem. Soc. 2005; 127:12442–12443. 16144363 10.1021/ja0529788

[175] Paige J.S., Wu K.Y., Jaffrey S.R. RNA mimics of green fluorescent protein. Science. 2011; 333:642–646. 21798953 10.1126/science.1207339 PMC3314379

[176] Warner K.D., Chen M.C., Song W., Strack R.L., Thorn A., Jaffrey S.R., Ferré-D’Amaré A.R. Structural basis for activity of highly efficient RNA mimics of green fluorescent protein. Nat. Struct. Mol. Biol. 2014; 21:658–663. 25026079 10.1038/nsmb.2865 PMC4143336

[177] Strack R.L., Disney M.D., Jaffrey S.R. A superfolding Spinach2 reveals the dynamic nature of trinucleotide repeat–containing RNA. Nat. Methods. 2013; 10:1219–1224. 24162923 10.1038/nmeth.2701 PMC3852148

[178] Paige J.S., Nguyen-Duc T., Song W., Jaffrey S.R. Fluorescence imaging of cellular metabolites with RNA. Science. 2012; 335:1194–1194. 22403384 10.1126/science.1218298 PMC3303607

[179] Bhadra S., Ellington A.D. A Spinach molecular beacon triggered by strand displacement. RNA. 2014; 20:1183–1194. 24942625 10.1261/rna.045047.114 PMC4105745

[180] Song W., Strack R.L., Jaffrey S.R. Imaging bacterial protein expression using genetically encoded RNA sensors. Nat. Methods. 2013; 10:873–875. 23872791 10.1038/nmeth.2568 PMC3758421

[181] Pothoulakis G., Ceroni F., Reeve B., Ellis T. The spinach RNA aptamer as a characterization tool for synthetic biology. ACS Synth. Biol. 2014; 3:182–187. 23991760 10.1021/sb400089c

[182] Rogers T.A., Andrews G.E., Jaeger L., Grabow W.W. Fluorescent monitoring of RNA assembly and processing using the split-spinach aptamer. ACS Synth. Biol. 2015; 4:162–166. 24932527 10.1021/sb5000725

[183] Kikuchi N., Kolpashchikov D.M. A universal split spinach aptamer (USSA) for nucleic acid analysis and DNA computation. Chem. Commun. 2017; 53:4977–4980. 10.1039/c7cc01540b 28425510

[184] Wang Z., Luo Y., Xie X., Hu X., Song H., Zhao Y., Shi J., Wang L., Glinsky G., Chen N. et al. In situ spatial complementation of aptamer-mediated recognition enables live-cell imaging of native RNA transcripts in real time. Angew. Chem. Int. Ed. 2018; 57:972–976. 10.1002/anie.201707795 28991414

[185] Bertucci A., Porchetta A., Ricci F. Antibody-templated assembly of an RNA mimic of green fluorescent protein. Anal. Chem. 2018; 90:1049–1053. 29131585 10.1021/acs.analchem.7b02102

[186] Strack R.L., Disney M.D., Jaffrey S.R. A superfolding Spinach2 reveals the dynamic nature of trinucleotide repeat–containing RNA. Nat. Methods. 2013; 10:1219–1224. 24162923 10.1038/nmeth.2701 PMC3852148

[187] Filonov G.S., Moon J.D., Svensen N., Jaffrey S.R. Broccoli: rapid selection of an RNA mimic of green fluorescent protein by fluorescence-based selection and directed evolution. J. Am. Chem. Soc. 2014; 136:16299–16308. 25337688 10.1021/ja508478x PMC4244833

[188] Halman J.R., Satterwhite E., Roark B., Chandler M., Viard M., Ivanina A., Bindewald E., Kasprzak W.K., Panigaj M., Bui M.N. et al. Functionally-interdependent shape-switching nanoparticles with controllable properties. Nucleic Acids Res. 2017; 45:2210–2220. 28108656 10.1093/nar/gkx008 PMC5389727

[189] Chandler M., Lyalina T., Halman J., Rackley L., Lee L., Dang D., Ke W., Sajja S., Woods S., Acharya S. et al. Broccoli fluorets: split aptamers as a user-friendly fluorescent toolkit for dynamic RNA Nanotechnology. Molecules. 2018; 23:3178. 10.3390/molecules23123178 PMC6321606 30513826

[190] Alam K.K., Tawiah K.D., Lichte M.F., Porciani D., Burke D.H. A fluorescent split aptamer for visualizing RNA–RNA assembly *in vivo*. ACS Synth. Biol. 2017; 6:1710–1721. 28548488 10.1021/acssynbio.7b00059 PMC5603824

[191] Torelli E., Kozyra J.W., Gu J.-Y., Stimming U., Piantanida L., Voïtchovsky K., Krasnogor N. Isothermal folding of a light-up bio-orthogonal RNA origami nanoribbon. Sci. Rep. 2018; 8:6989. 29725066 10.1038/s41598-018-25270-6 PMC5934368

[192] Karunanayake Mudiyanselage A.P.K.K., Yu Q., Leon-Duque M.A., Zhao B., Wu R., You M. Genetically encoded catalytic hairpin assembly for sensitive RNA imaging in live cells. J. Am. Chem. Soc. 2018; 140:8739–8745. 29944357 10.1021/jacs.8b03956 PMC6201751

[193] Toran P., Smolina I., Driscoll H., Ding F., Sun Y., Cantor C.R., Broude N.E. Labeling native bacterial RNA in live cells. Cell Res. 2014; 24:894–897. 24732010 10.1038/cr.2014.47 PMC4085757

[194] Ma X., Guo Z., Mao Z., Tang Y., Miao P. Colorimetric theophylline aggregation assay using an RNA aptamer and non-crosslinking gold nanoparticles. Microchim. Acta. 2018; 185:33. 10.1007/s00604-017-2606-4 29594625

[195] Müller S., Appel B., Balke D., Hieronymus R., Nübel C. Thirty-five years of research into ribozymes and nucleic acid catalysis: where do we stand today? [version 1; peer review: 2 approved]. F1000Research. 2016; 5:1511. 10.12688/f1000research.8601.1 PMC4926735 27408700

[196] Hampel A., Tritz R. RNA catalytic properties of the minimum (-)sTRSV sequence. Biochemistry. 1989; 28:4929–4933. 2765519 10.1021/bi00438a002

[197] Welz R., Schmidt C., Müller S. Spermine supports catalysis of hairpin ribozyme variants to differing extents. Biochem. Biophys. Res. Commun. 2001; 283:648–654. 11341774 10.1006/bbrc.2001.4829

[198] Sullenger B.A., Cech T.R. Ribozyme-mediated repair of defective mRNA by targeted trans-splicing. Nature. 1994; 371:619–622. 7935797 10.1038/371619a0

[199] Hasegawa S., Gowrishankar G., Rao J. Detection of mRNA in mammalian cells with a split ribozyme reporter. ChemBioChem. 2006; 7:925–928. 16671127 10.1002/cbic.200600061

[200] Gao W., Xing B., Tsien R.Y., Rao J. Novel Fluorogenic substrates for imaging β-lactamase gene expression. J. Am. Chem. Soc. 2003; 125:11146–11147. 16220906 10.1021/ja036126o

[201] Wang R., Zhang Q., Zhang Y., Shi H., Nguyen K.T., Zhou X. Unconventional split aptamers cleaved at functionally essential sites preserve biorecognition capability. Anal. Chem. 2019; 91:15811–15817. 31625719 10.1021/acs.analchem.9b04115

[202] Li Q., Wang Y.-D., Shen G.-L., Tang H., Yu R.-Q., Jiang J.-H. Split aptamer mediated endonuclease amplification for small-molecule detection. Chem. Commun. 2015; 51:4196–4199. 10.1039/c5cc00390c 25672262

[203] He J.-L., Wu Z.-S., Zhou H., Wang H.-Q., Jiang J.-H., Shen G.-L., Yu R.-Q. Fluorescence aptameric sensor for strand displacement amplification detection of cocaine. Anal. Chem. 2010; 82:1358–1364. 20078091 10.1021/ac902416u

